# An In Vitro Screen Identifies H1 Influenza Hemagglutinin Substitutions That Alter mRNA-LNP Vaccine Responses Against the Stalk Domain

**DOI:** 10.3390/vaccines14080701

**Published:** 2026-08-13

**Authors:** Samuel W. Rovito, Po-Ling Chen, Guohua Yang, Haley N. Writt, Ashley N. Zalla, Marissa A. Donofrio, Jonathan N. Fogo, Richard J. Webby, James D. Brien, Charles J. Russell

**Affiliations:** 1 St. Jude Graduate School of Biomedical Sciences, St. Jude Children’s Research Hospital, Memphis, TN 38105, USA; samuel.rovito@stjude.org (S.W.R.); richard.webby@stjude.org (R.J.W.); 2 Department of Host–Microbe Interactions, St. Jude Children’s Research Hospital, Memphis, TN 38105, USA; po-ling.chen@stjude.org (P.-L.C.); haley.writt@stjude.org (H.N.W.); 3 Department of Microbiology, Immunology, and Molecular Genetics, University of Kentucky College of Medicine, Lexington, KY 40536, USA; anzalla02@gmail.com (A.N.Z.);; 4 Department of Microbiology, Immunology, and Biochemistry, University of Tennessee Health Science Center, Memphis, TN 38163, USA

**Keywords:** influenza vaccine, hemagglutinin, mRNA vaccine, H1N1, immunogenicity, antigen engineering, stalk antibody

## Abstract

**Background:** Conformational stability has been shown to modulate the immunogenicity of structural class 1 viral fusion glycoproteins, yet the relationship between influenza A hemagglutinin (HA) stability and antigenicity remains poorly understood. **Methods:** Here, we screened a panel of 21 A/Hawaii/70/2019 (H1N1) HA mutants for expression, cleavage, and acid and conformational stability in cells. Twelve mutant HA proteins expressed from transfected plasmid constructs that showed relatively robust expression compared to WT also exhibited either altered stability or glycosylation deletion. mRNA-LNPs were generated containing these 12 HAs, along with the wild-type (WT) HA, to investigate the mutational effects on mRNA-LNP vaccine immunogenicity and protection in mice. Hemagglutination inhibition, microneutralization, and total serum IgG ELISAs were performed using a panel of H1N1 antigens to evaluate humoral immune responses at 28 days post vaccination. The vaccinated mice were then subjected to lethal challenge with a forward-drifted H1N1 virus. **Results:** Most mutant HA candidates elicited WT-like serological responses and provided protection against challenge, although noticeable decreases in serological responses and, to a lesser extent, protection were observed, especially among G72K- and R109E-vaccinated mice. Of note, substitutions at position E107 enhanced total IgG responses against the HA stalk. Modest, but detectable, increases in antibody-dependent cellular cytotoxicity (ADCC) were also observed, particularly with E107R. Although substitution-specific differences in weight loss were found in E107-vaccinated mice challenged with a mouse-adapted Cal/09 virus, these differences were minor, and protection against the forward-drifted challenge virus and a heterologous PR8 challenge virus was no different from that in mice vaccinated with WT HA. **Conclusions:** These findings demonstrate the ability of specific substitutions to alter components of humoral immunity by shifting HA domain-specific antibody responses, which could prove useful in the design and development of HA vaccine antigens possessing optimized immunogenicity.

## 1. Introduction

Seasonal influenza vaccine effectiveness varies between 10% and 60% annually because of factors including antigenic drift, vaccine antigen mismatch, and production-associated mutations [1,2]. Currently, licensed influenza vaccines in the United States are produced in the form of inactivated viruses, live attenuated viruses, and recombinant proteins. Adjuvants and high-dosage strategies have also been used to facilitate dose sparing and improved protection in vulnerable populations [3,4]. Next-generation vaccine platforms such as the mRNA-LNP platform have recently been approved by the European Medicines Agency [5]. The hemagglutinin (HA) is the immunodominant target of current vaccines [6], and is structurally divided into a membrane-distal head domain, consisting of HA1 residues, and a relatively conserved membrane-proximal stalk domain, comprising HA2 and HA1 terminal residues. In general, HA sequences are diverse and are largely tolerant of mutations within the head domain, thus driving immune escape and necessitating regular vaccine reformulation. Improving anti-HA immunity is the subject of intense research, as it may enable the development of therapeutics that will protect against a wide breadth of influenza viruses.

Influenza A virus HA proteins are homotrimeric surface class 1 fusion glycoproteins encompassing 18 known subtypes (H1–H18) [7]. Specifically, H1 and H3 viruses circulate seasonally in humans. H1 and H3 HAs are synthesized as inactive precursor molecules (HA0) that require cleavage by trypsin-like proteases to mature into fusogenic forms consisting of HA1 and HA2 subunits. HA1 itself consists of the receptor-binding domain (RBD), the vestigial esterase domain (VED), and the fusion subdomain (FD). During infection, the RBD binds to sialic acid-containing cell receptors, enabling virion uptake by endocytosis. As the endosome acidifies, the HA undergoes drastic pH-dependent refolding (usually at a pH between 5.0 and 6.0) that drives fusion between the viral and host-cell membranes. HA conformational changes begin with the reversible release of the fusion peptide (FP) from its pocket and the detachment of the head from the stalk in a process known as “breathing” [8]. On a scale of minutes, a fully extended intermediate forms irreversibly, coinciding with FP insertion into the target membrane. The FP and membrane-proximal regions are then brought together in a stable post-fusion hairpin, bringing the membranes into proximity for fusion [8,9,10]. The relative ease with which an HA undergoes a conformation change upon exposure to mild acid is termed HA stability. Relatively stable HA proteins exhibit greater resistance to acid-induced conformation changes when compared with less stable HAs [11]. HA stability contributes to viral host range, zoonosis, and pandemic potential [12].

Current seasonal influenza vaccines typically elicit narrow, strain-specific protective immunity directed against the immunodominant, but relatively variable, HA head [13]. However, efforts have been made to improve anti-HA protective breadth. One broad strategy seeks to engineer antigens that enhance humoral immunity against conserved epitopes, employing approaches such as chimeric HA prime-boost regimens, glycosylation shielding, stalk-only HAs, and “mosaic” antigens [14,15,16,17,18]. Indeed, structural studies continue to identify conserved epitopes targeted by broadly reactive antibodies [19]. Strategies involving computationally optimized broadly reactive antigens (COBRAs) have also shown early promise in eliciting broadly protective responses; they use consensus-building algorithms to generate antigens possessing conserved linear epitopes within the head [20,21,22,23]. Novel, next-generation viral-vectored vaccine platforms, such as adeno-associated virus (AAV)-based and PIV5-based approaches, have also shown early promise in eliciting greater reactive breadth [22,24].

Relations between class 1 fusion protein stability and antigenicity have been demonstrated. For example, substitutions in the SARS-CoV-2 spike protein that stabilize its pre-fusion conformation have been useful in developing highly protective, successful vaccines [25]. Stabilizing the pre-fusion conformation of the RSV F-protein has been instrumental in producing protective vaccines used in elderly and high-risk groups [26]. However, stability–antigenicity relations in the influenza HA protein are less clear. Anti-HA antibodies showing conformation-specific binding have been reported [27], suggesting that HA conformational states influence epitope presentation. Interestingly, novel broadly reactive epitopes have also been found at spatially occluded interfaces between head protomers, implying a relation between HA conformation and epitope availability [28,29,30]. Conversely, a pan-H3 protective antibody was found to span the VED and RBD in the head [31], suggesting that optimal stability between HA subdomains enhances the integrity of similar epitopes. However, locking HA in the pre-fusion conformation by ablating the trypsin cleavage site did not alter HA humoral immunity, suggesting that HA fusogenicity does not affect antigenicity [32]. Additionally, recent studies have obtained antigenic benefits by stabilizing HA2 [33,34]. Directly assessing the relation between HA stability and antigenicity would help to contextualize these findings and provide clarity about HA antigenic behavior.

Nucleoside-modified messenger RNA lipid nanoparticle (mRNA-LNP) vaccines first demonstrated their safety and effectiveness against SARS-CoV-2 by showing the superior immunogenicity of a stabilized spike protein antigen [25,35]. mRNA-LNPs have also been used extensively in influenza vaccine development [35,36], with several studies assessing engineered HA antigens with mRNA-LNPs [21,32,37]. Like viral vector vaccines, mRNA-LNPs can deliver and express extensively engineered antigens with ablated native function (i.e., a lack of fusogenicity [38]). Furthermore, they do not require virus production or pose a risk of genome integration [39]. This capability of the mRNA-LNP platform is shared with the recombinant protein vaccine platform, which also requires the expression and purification of variant HA proteins. In addition, mRNA-LNP vaccines support the convenient direct interchange of HA modifications between plasmid constructs used to screen antigens and those used for vaccine synthesis. Given these properties, mRNA-LNP vaccines are well suited to investigating complex relationships such as that between HA stability and antigenicity.

In a previous study, most H1N1 HA mutations that increased acid stability provided no notable enhancements in mRNA-LNP immunogenicity or heterosubtypic protection in a mouse model [34]. Therefore, in the present study, we produced a broader panel of 21 HA modifications, focusing on residues previously shown or predicted to alter HA head domain conformational stability, head–stalk interaction, or glycosylation. We first used pCAGGS plasmid-expressed HA proteins as a cost-effective in vitro screen to evaluate expression, cleavage, cell-surface display, and stability phenotypes before advancing 12 constructs into mRNA-LNP vaccine formulations. This screen-to-vaccine strategy identified substitutions at HA1 position 107 as a particularly informative antigen-engineering site. E107 substitutions increased antibody responses against the HA stalk, modestly improved ADCC activity against H1N1 antigens, and maintained WT-like protection across multiple mouse challenge models.

## 2. Materials and Methods

### 2.1. Cell Lines and Viruses

HEK-293T, Vero, and MDCK cells (ATCC, Manassas, VA, USA) were grown in culture at 37 °C in 5% CO_2_ in, respectively, OptiMEM, DMEM, and MEM (Thermo Fisher, Waltham, MA, USA). The medium was supplemented with 10% fetal bovine serum (Cytiva, Wilmington, DE, USA) and 1% penicillin–streptomycin (Thermo Fisher). A virus with A/Hawaii/70/2019(H1N1; HI/19) HA and NA, as well as A/Puerto Rico/8/1934 (H1N1; PR8) internal gene segments, was generated by reverse genetics [27]. Viral stocks were grown in MDCK cells and quantified as described previously [27,40]. Other H1N1 viruses used in the study included wild-type (WT) A/Hawaii/70/2019 (Hawaii/19), A/Victoria/4897/2022 (Vic/22), A/Michigan/45/2015 (Mich/15), A/California/04/2009 (Cal/09), mouse-adapted A/California/04/2009 (MA-Cal/09), and A/Puerto Rico/8/1934 (PR8).

### 2.2. Hemagglutinin Substitution Selection, Mutagenesis, and Vector Preparation

The A/Hawaii/70/2019 (H1N1) HA cDNA sequence was retrieved from the GISAID initiative (EPI_SET_250616kp, https://doi.org/10.55876/gis8.250616kp) and codon-optimized for expression in human cells; https://www.idtdna.com/pages/tools/codon-optimization-tool (accessed on 8 January 2024). HA DNA was synthesized (Twist Bioscience, San Francisco, CA, USA) and subcloned into the pCAGGS expression vector [41], using Phusion™ High-Fidelity PCR Master Mix (New England Biolabs, Ipswich, MA, USA) in accordance with the manufacturer’s instructions. Substitutions were introduced based on previous studies and by examining the Cal/09 HA structure in PyMol v2.4.0 (PDB code 3UBE) [17,27,42,43,44,45,46,47,48,49,50,51]. Site-directed mutagenesis was conducted using QuikChange II Kits (Agilent, Santa Clara, CA, USA) in accordance with the manufacturer’s instructions. Plasmid stocks were grown in DH10B cells (Thermo Fisher), prepared with a Qiagen Plasmid Maxi Kit (Qiagen, Venlo, The Netherlands), and sequence-confirmed by Sanger sequencing. Some plasmid constructs were synthesized, cloned, and prepared commercially (Twist Bioscience; GenScript, Nanjing, China).

### 2.3. Preparation of Nucleoside-Modified mRNA Transcripts and mRNA-LNPs

Codon-optimized HI/19 HA mutant genes were cloned as described above into a pJB_pUC57 plasmid possessing a T-7 polymerase promoter, a 5′ UTR (AGAATACAAGCTACTTGTTCTTTTTGCA), a 3′ UTR, a poly(A) tail, and a BspQI restriction site. Plasmid templates were digested with BspQI (New England Biolabs) according to the manufacturer’s protocol. Capped mRNA transcripts were then synthesized using a HiScribe T7 mRNA Kit with CleanCap Reagent (New England Biolabs) and pseudouridine-5′-triphosphate (TriLink, San Diego, CA, USA). mRNA-LNPs were formulated using GenVoy-ILM lipid mixture (Cytiva). mRNA was dissolved in 50 mM acetic acid (pH 4.0) and mixed at 12 mL/min, in a 3:1 volume ratio, with ILM diluted 1:1 in 100% ethanol, using a microfluidics LNP formulation system on a 490 Trident T chip (Sunshine, Unchained Labs, Pleasanton, CA, USA). Particle diameter and PDI were measured using the Stunner plate-based dynamic light-scattering instrument (Unchained Labs). Zeta potential was measured using a Malvern Zetasizer Lab (Malvern Panalytical, Malvern, Worcestershire, U.K). For zeta-potential measurements, mRNA-LNP samples were diluted 1:10 in sterile 10 mM NaCl, pH 6.5, and transferred to a capillary measurement cell. Samples stored at 4 °C were diluted in room-temperature diluent and allowed to equilibrate to room temperature before measurement. The RNA content and encapsulation efficiency were assessed with an Invitrogen Quant-iT RiboGreen RNA Assay Kit (Thermo Fisher), used in accordance with the manufacturer’s protocols with minor modifications, where total RNA was measured with and without triton-X100 to measure the quantity of mRNA encapsulated. mRNA-LNPs were dialyzed in PBS with Ca^2+^ and Mg^2+^ (PBS^+/+^), centrifugally concentrated at a 10-kD molecular weight cut-off (MWCO) (MilliporeSigma, Burlington, MA, USA), filtered with 0.2 µm syringe filters (Cytiva), and stored at 4 °C.

### 2.4. Transfection of HA Plasmids and HA mRNA Transcripts In Vitro

HEK-293T cells were seeded at 1,000,000, 400,000, or 150,000 cells per well in 6-, 12-, and 24-well tissue culture-treated plates, respectively. Vero cells were seeded at 250,000, 150,000, or 65,000 cells per well in 6-, 12-, and 24-well plates, respectively. After incubation for 24 h at 37 °C in 5% CO_2_, the monolayers were transfected with 3 µg, 1 µg, or 0.5 µg, respectively, of HA plasmid, using Lipofectamine 3000 (Thermo Fisher), and incubated as above according to the manufacturer’s protocol.

### 2.5. Total Cellular Expression of HA Plasmids and HA mRNA Transcripts In Vitro

In vitro expression was performed as described [12]. Briefly, transfected HEK-293T cells were treated with 5 µg/mL TPCK-treated trypsin for 15 min at 37 °C, washed with PBS, Ca2^+^, and Mg2^+^ (PBS^+/+^), and lysed for 30 min in radioimmunoprecipitation buffer containing cOmplete Mini EDTA-free Protease Inhibitor Cocktail tablets (Roche, Basel, Switzerland). In each case, a 12 µg aliquot of total protein was boiled for 7 min under reducing conditions, then subjected to electrophoresis at 150 V for 90 min on a NuPAGE Bis-Tris 4–12% PAGE gel (Thermo Fisher). The proteins were then transferred to PVDF membranes at 25 V for 90 min. Blots were stained with G.618 anti-A/California/04/2009(H1N1) polyclonal goat serum (diluted 1:1000) (BEI Resources, Manassas, VA, USA; catalog no. NR15696) and anti-goat IgG-HRP antibody (diluted 1:2000) (Thermo Fisher), then imaged with a Li-Cor Odyssey XF system (Licor Bio, Lincoln, NE, USA). β-Actin was identified with a mouse anti-β-actin antibody (diluted 1:2000) (Santa Cruz Biotechnology, Dallas, TX, USA) and an anti-mouse IgG-HRP antibody (diluted 1:2000) (Cell Signaling Technology, Danvers, MA, USA). Mean gray values were analyzed with ImageJ v1.54r software and normalized to WT [52]. The bands present in parallel gels within one experiment represent the same sample. Total HA expression was defined as the total of HA0 + HA1 + HA2. The percentage of HA cleaved by TPCK-trypsin was defined as (HA1 + HA2)/(HA0 + HA1 + HA2) × 100.

### 2.6. HA Cell-Surface Expression by Flow Cytometry

Flow cytometry was performed as described previously [27]. Briefly, transfected HEK-293T monolayers were scraped and suspended in the transfection media, then washed twice with ice cold FACS buffer (PBS^−/−^, 1% BSA), and stained in 50 µL of FACS buffer containing 0.5 µg of CR6261 anti-HA monoclonal antibody (mAb) (Creative Biolabs, Shirley, NY, USA) and isotype control (Thermo Fisher) for 1 h at 4 °C. Samples were stained with goat anti-human IgG–Alexa Fluor 594 secondary antibody (diluted 1:500) (Thermo Fisher) for 30 min at 4 °C in the dark, rinsed, and suspended in 300 µL of FACS buffer plus 1 µg/mL 4′,6-diamidino-2-phenylindole (DAPI) (Thermo Fisher). For each sample, the geometric mean fluorescence (GMF) of 5000 live single cells was assessed using an LSRFortessa Cell Analyzer and FlowJo v10.10.0 software (BD Biosciences, East Rutherford, NJ, USA).

### 2.7. pH-Buffered Saline for HA Stability Assays

The pH of PBS^+/+^ was adjusted to pH values between 6.8 and 5.0 at 0.1 or 0.2 pH-unit intervals by using 0.1 M citric acid in ddH_2_O.

### 2.8. pH of Activation by Syncytia Formation Assay

HA functional stability was assessed [27]. Transfected Vero cells were washed twice with PBS^+/+^, treated with DMEM + 5 µg/mL TPCK trypsin for 15 min at 37 °C, then neutralized with DMEM + 10% FBS. Monolayers were washed as described above, overlaid with pH-adjusted PBS^+/+^ for 15 min at 37 °C, then incubated with DMEM + 10% FBS at 37 °C for 3 h. Cells were fixed and stained with a Hema 3 Stat Pack (Fisher Scientific, Pittsburgh, PA, USA) according to the manufacturer’s protocols and photographed using brightfield microscopy. The pH of activation is defined as the highest pH value at which syncytia form.

### 2.9. pH of Conformational Change Assay Using FluA-20 Monoclonal Antibody

A series of six transfected Vero cell samples were suspended in DMEM, then treated with 5 µg/mL TPCK-treated trypsin for 15 min at 37 °C. Next, the cells were treated with PBS^+/+^ that was pH-buffered to within pH 6.6–5.0 at 0.2 pH-unit intervals for 10 min at 37 °C. Each cell sample was treated with 0.5 µg of FluA-20 anti-HA mAb (Creative Biolabs) in 50 µL of FACS buffer for 1 h at 4 °C, then with Goat anti-human IgG–Alexa Fluor 594 antibody (diluted 1:500) (Thermo Fisher) for 30 min at 4 °C in the dark [29]. Cells were suspended in FACS buffer + 1 µg/mL DAPI. The net GMF values of 2000 live singlets per sample were determined and analyzed by subtracting the isotype control GMFs from the sample GMFs. The fluorescence change within each series was quantified by subtracting the minimum observed GMFs from the maximum GMFs. To find the pH of conformation change, GMFs in a series were normalized as the percentage of the total fluorescence change in that series, and outliers were identified and removed by the false discovery rate (Q = 1%) [53]. Then, data were fitted to four-parameter sigmoidal dose–response curves by unweighted, nonlinear, least-squares regression. The pH of conformation change was defined as the pH resulting in 50% GMF change. The stability-altering substitution, H26W, was similarly fitted to an exponential growth model.

### 2.10. Mouse Immunization and Challenge

Groups of 6- to 8-week-old female DBA/2J mice (Jackson Laboratory, Bar Harbor, ME, USA) (*n* = 5 mice per group) were immunized by intramuscular injection of 0.5 µg of HA mRNA-LNPs in a total volume of ≤100 µL of mRNA-LNP in PBS^−/−^ delivered as ≤50 µL per hamstring. Standard-of-care controls were injected with Flucelvax quadrivalent inactivated vaccine (QIV), Fall 2020–2021, at 1.5 µg of pH1N1 HA per mouse (CSL-Seqirus Melbourne, Australia). Negative-control mice were given 1 µg of poly(A) mRNA-LNP or PBS^−/−^. All immunizations were performed using 30G U-100 insulin syringes (BD Biosciences). Repeat experiments yielded a total of *n* = 10 to *n* = 15 mice per treatment group per virus challenge. Blood was collected submandibularly at day 28 post vaccination, incubated at 4 °C overnight, and centrifuged for serum collection. Mice were challenged by intranasal inoculation with 10× the median lethal dose 50% (mLD_50_) of Vic/22 (3.3 × 10^4^ PFU/mouse), PR8 (100 TCID_50_/mouse), or MA-Cal/09 (2.3 × 10^4^ TCID_50_/mouse) on day 29 or day 33 post vaccination. Weight and survival were monitored for 14 days, using 25% weight loss as the humane endpoint. In a separate experiment, *n* = 4 or *n* = 5 mice were humanely euthanized at day 3 post challenge and their lungs were collected, homogenized with a TissueLyser II bead-mill homogenizer (Qiagen), centrifuged at 12,000 rpm for 15 min at 4 °C, aliquoted, then frozen at −80 °C. The viral load was then determined in TCID_50_/mL. Cages, food, and water were changed and refreshed one to two times weekly.

### 2.11. Hemagglutination Inhibition (HI) and Microneutralization (MN) Assays

HI and MN assays were performed as described previously [40]. For HI assays, sera were treated with Receptor Destroying Enzyme II (Hardy Diagnostics, Santa Maria, CA, USA) according to the manufacturer’s protocol and serially diluted 2-fold, starting at 1:20. This was followed by the addition of 4 hemagglutination units (HAU) of virus per well and incubation at room temperature (RT) for 1 h. A 0.5% suspension of turkey red blood cells was added to each well (in a 1:1 ratio by volume), and the wells were incubated for 30 min at RT. MN assays used 2-fold serially diluted RDE-treated sera, starting with a 1:40 dilution. For each assay, 100 TCID_50_ of virus were added to each well, the plates were incubated at 37 °C for 1 h, and the results were validated by titration. Inoculum was overlaid in technical triplicate onto 30,000 MDCK cells per well in 96-well plates, which were then incubated at 37 °C for 1 h. The inoculum was then replaced with DMEM + 1 µg/mL TPCK-treated trypsin, and the plates were incubated for 72 h at 37 °C in 5% CO_2_. HI titers corresponded to the highest reciprocal dilution that did not result in hemagglutination; MN titers were read by HA assay as the highest reciprocal dilution that did not result in hemagglutination in at least two of three replicates. HI titers at and above 40 are considered seroprotective [54].

### 2.12. Preparation of Inactivated Viral Antigens and Recombinant Protein Reagents

β-Propiolactone (MilliporeSigma) and NaOH were added to harvested viral stocks at final concentrations of 0.05% and 500 µM, respectively, and the mixture was rocked overnight at 4 °C. NaHCO_3_ was then added to 0.075%, the mixture was incubated for 1 h at 37 °C, and the pH was re-adjusted to 7.0. Tangential flow filtration, using Pellicon XL 50 filtration cassettes and Ultracel 1000 kDa nominal molecular weight cutoff (NMWCO) membranes (MilliporeSigma), was performed on the inactivated viruses according to the manufacturer’s protocols. Antigens were validated by Bradford assays (Thermo Fisher) and HA assays. “Foldon” trimerization domains and His6 tags were added to the C-terminals of two previously engineered HA sequences. A chimeric HA was used that possessed an A/Mallard/Sweden/81/2002 (H6N1) head and an A/California/04/2009 (H1N1) stalk. A stalk-only antigen, MiniHA #4900, contained the stalk domain of A/Brisbane/59/2007 (H1N1) [15,55,56]. Sequences were synthesized and cloned (GenScript) into pAcGP67A plasmids for Bac-to-Bac baculovirus protein expression, which was performed in accordance with the manufacturer’s user guide (Thermo Fisher; MAN0000414). Purification was performed by Ni2^+^ affinity chromatography as described previously [57].

### 2.13. Enzyme-Linked Immunosorbent Assays

ELISAs were performed as described previously [37]. Briefly, MaxiSorp 96-well plates (Thermo Fisher) were coated with 0.2 µg/mL antigen overnight, washed with PBS^−/−^ + 0.1% Tween 20 (PBST), and blocked with PBST + 1.5% BSA. Sera were serially diluted 5-fold for the total IgG antigen panel and 4-fold for chimeric and MiniHA ELISAs, with starting dilutions of 1:80, 1:20, and 1:20, respectively, and incubated for 1 h at RT. Samples were then stained with goat anti-mouse IgG-HRP polyclonal antibodies (Thermo Fisher) (diluted 1:7500) for 1 h at RT in the dark. TMB ELISA substrate was applied according to the manufacturer’s protocol (Thermo Fisher), and the absorbance (at 450 nm) was read on a BioTek Synergy H1 plate reader (Agilent). Cutoff values were averages of values for blank control wells plus three SDs. Endpoint titers were defined as the highest reciprocal dilution with absorbance greater than the cutoff.

### 2.14. ADCC Reporter Assays

ADCC reporter assays (Promega, Madison, WI, USA) were performed according to the manufacturer’s directions. Madine–Darby canine kidney (MDCK) cells (1 × 10^4^ cells per well) were plated on tissue culture-treated white, flat-bottom 96-well plates (Corning, Corning, NY, USA) and allowed to double at 37 °C in 5% CO_2_ overnight. The next day, the cells were washed with PBS^+/+^, then infected at an MOI of 0.2 with PR8, Cal/09, Mich/15, Hawaii/19, or Vic/22 virus, using DMEM + 1 µg/mL TPCK-treated trypsin. Infections were allowed to progress overnight at 37 °C in 5% CO_2_. The inoculum was then removed and replaced with 25 µL of assay buffer (RPMI 1640 + 4% low-IgG FBS) (Thermo Fisher) per well. Day 28 post-vaccination sera from vaccination groups (*n* = 5 per group) were pooled, then treated at 56 °C for 30 min. The sera from three different groups were assessed. Sera were serially diluted 3-fold in assay buffer, with an initial dilution factor of 1:5, then 25 µL aliquots of diluted sera were added to the cells in technical triplicate, and the plates were incubated at 37 °C for 30 min. ADCC effector Jurkat cells expressing murine FcγRIV linked to an NFAT-activated luciferase reporter (Promega) were added to each well at 7.5 × 10^4^ cells/well. The serum dilution was now 1:15. The reaction mixtures were incubated at 37 °C in 5% CO_2_ for 6 h, then 75 µL/well of Bio-Glo luciferase assay reagent was added and the luminescence was quantified on a BioTek Synergy H1 plate reader (Agilent). The fold induction was calculated in relative light units (RLUs), defined as (RLU_sample_ − RLU_background_)/(RLU_no-sera controls_ − RLU_background_), and plotted as a function of the dilution factor. Areas under the curves were then calculated.

### 2.15. Statistical Analyses

Statistical analyses were performed using GraphPad Prism v10.4.0 (Dotmatics, Boston, MA, USA). Significance is shown as * *p* < 0.05, ** *p* < 0.01, *** *p* < 0.001, or **** *p* < 0.0001. All flow cytometry data were analyzed using FlowJo v10.10.0 software (BD Biosciences). Statistical comparisons were made only between data sets that included data points above the threshold/limit of detection. Total cellular expression, cell-surface protein expression, and the percentage of cleaved HA were compared for significance by one-way ANOVA with Tukey’s multiple comparisons correction test. The pH of activation was compared to that of the WT HA by one-way ANOVA with Dunnett’s multiple comparisons correction test. An unpaired Welch’s *t*-test was used to compare FluA-20 mAb binding between pH = 7.0 and pH = 5.0. To find the pH of conformation change, outliers were first identified by false-discovery rate (Q = 1%) [53]. Data were then fitted to four-parameter sigmoidal dose–response curves by unweighted, nonlinear least-squares regression. H26W was similarly fitted to an exponential growth model. HI, MN, IgG ELISA, Vic/22 maximum weight loss, chimeric HA ELISA, MiniHA ELISA, and viral load (TCID_50_/mL) data were all compared to WT data by using a Kruskal–Wallis test with Dunn’s multiple comparisons correction test. ADCC data were compared to WT data by two-way ANOVA with Dunnett’s multiple comparisons correction test. Per diem weight loss was compared to that in mice vaccinated with WT HA through the last timepoint at which at least one mouse in each group (*n* = 5) was still alive. This was done by using a matched mixed-effects analysis with a Geisser–Greenhouse variability correction and Dunnett’s multiple comparisons correction. To ensure comparison of groups with at least one mouse each, weight loss in response to PR8, MA-Cal/09, and Vic/22 challenges was compared, respectively, through days 6, 7, and 7 post infection. Survival was compared individually to that of the WT-vaccinated mice by log-rank tests.

## 3. Results

### 3.1. Selection of HA Substitutions for In Vitro Screening

**Figure 1:**
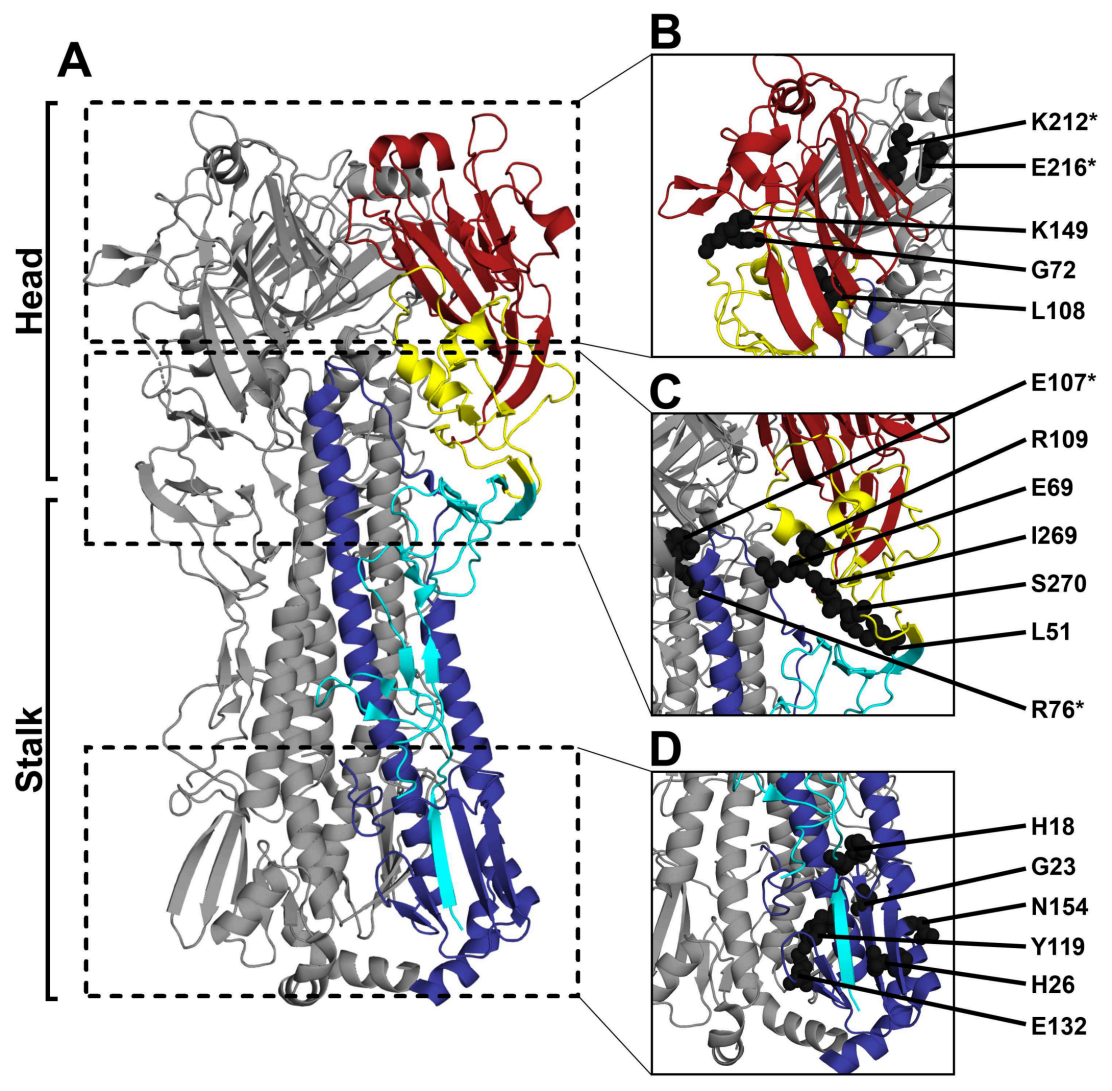
H1N1 HA structure and locations of residues containing substitutions. (**A**) Cal/09 H1 structure, with HA2 in dark blue. HA1 fragment subdomains are the fusion domain (FD) in cyan, the vestigial esterase domain (VED) in yellow, and the receptor-binding domain (RBD) in red. (**B**–**D**) Substitutions, shown in black. (**B**) Screened HA-head substitutions putatively alter the stability of the interface between the VED and RBD and between RBDs. (**C**) Head–stalk mutations putatively alter interactions between the VED and the B-loop, C-helix, and FD. (**D**) Stalk substitutions putatively stabilize the fusion peptide pocket, histidine switch 1, and D-helix contacts. N154S is predicted to ablate glycosylation. * Inter-protomeric residues. PDB: 3UBE [64].

A test set of 21 A/Hawaii/70/2019 (HI/19) HA constructs containing single or double amino acid substitutions anticipated to alter HA stability were introduced into the pCAGGS expression vector (Figure 1A; Table 1). Residues are hereafter denoted by H3 (HA1 residues) and HA2 numbering. Substitutions were both designed *de novo* and selected based on prior studies [17,27,42,44,45,46,47,48,49,50,51]. The in vitro phenotypes of these plasmid-expressed, full-length, membrane-bound mutant HAs were then examined at 24 h post transfection in common mammalian cell lines, as this timepoint likely coincides with maximal target protein expression but decreasing mRNA vaccine translation [58,59].

HA head stability was examined at the VED–RBD interface by introducing substitutions at G72, a conserved VED α-helix cap residue [60], and at K149 in the RBD (Figure 1B). G72C-K149C and G72D were, therefore, designed to form respective disulfide and salt bridge interactions with K149 [62], whereas G72I and G72K were designed to create a steric clash and electrostatic repulsion (Figure 1B; Table 1). L108K was introduced to disrupt VED 110-helix hydrophobic packing with the RBD. Additionally, a K212C-E216C disulfide bond was introduced to lock inter-protomeric hydrogen bonds between these residues [17,42], whereas E216K was selected to generate electrostatic repulsion [49]. None of these substitutions overlap with classical immunodominant H1 head epitopes [63].

Stability at the interface between the head and stalk domains was assessed by introducing a putative R109C-E69C disulfide bond to lock polar contacts between the VED and the HA2 B-loop (Figure 1C; Table 1) [49]. R109G and R109E were introduced to disrupt these contacts [49]. Inter-protomeric polar interactions were examined between E107 in the VED and E74 and R76 in HA2 by introducing E107C-R76C, E107A, and E107R to, respectively, form disulfide bonds, ablate polar contacts, and create electrostatic repulsion [43]. HA1 FD residue L51, although adjacent to the C52-C277 disulfide bond, is closely situated to VED residue S270 [51,60]. Therefore, L51C-S270C disulfide bonds and L51K-S270D salt bridges were designed to stabilize intra-HA1 head–stalk interactions. In contrast, I269G was designed to hinder inter-protomeric packing here, based on the effects of R269G in H3 [49].

Enhancing fusion-domain stability in other class 1 viral fusion glycoproteins is essential to generate vaccine-elicited immunity against these antigens [25,26]. Early data suggest that stabilizing certain regions within HA2 results in immunogenic benefits [33,34], but these effects remain poorly understood. To further explore this phenomenon, H18Q and G23C, known to stabilize the fusion peptide pocket in H5 and H3, were introduced (Figure 1D; Table 1) [27,45]. A putative Y119C-E132C disulfide bond was designed to stabilize interactions between structurally adjacent β-turn-β and D-helix features. H26W was introduced to stabilize a pH-sensitive histidine-switch region by forming π-cation interactions with K153 [48]. N154S was also included to ablate a known glycosylation site adjacent to K153 [46].

### 3.2. In Vitro Cellular Expression and Maturation of pCAGGS Plasmid-Expressed HA Proteins

Robust target antigen expression has been linked to mRNA-LNP vaccine immunogenicity [65]. We developed a screen-to-vaccine pipeline by utilizing the pCAGGS expression vector to screen the in vitro cellular expression profiles of the mutant HA antigens, as compared to the WT antigen, without necessitating mRNA vaccine transcript preparation of deleterious mutant HAs. HA substitutions identified as decreasing protein expression by more than 20% by any cellular expression metric when compared to the WT HA (*p* < 0.05) were excluded from downstream mRNA-LNP vaccine formulation and further evaluation. These mutants included G72C-K149C, G72I, L108K, L51C-S270C, L51K-S270D, G23C, and Y119C-E132C (Table 2).

**Figure 2:**
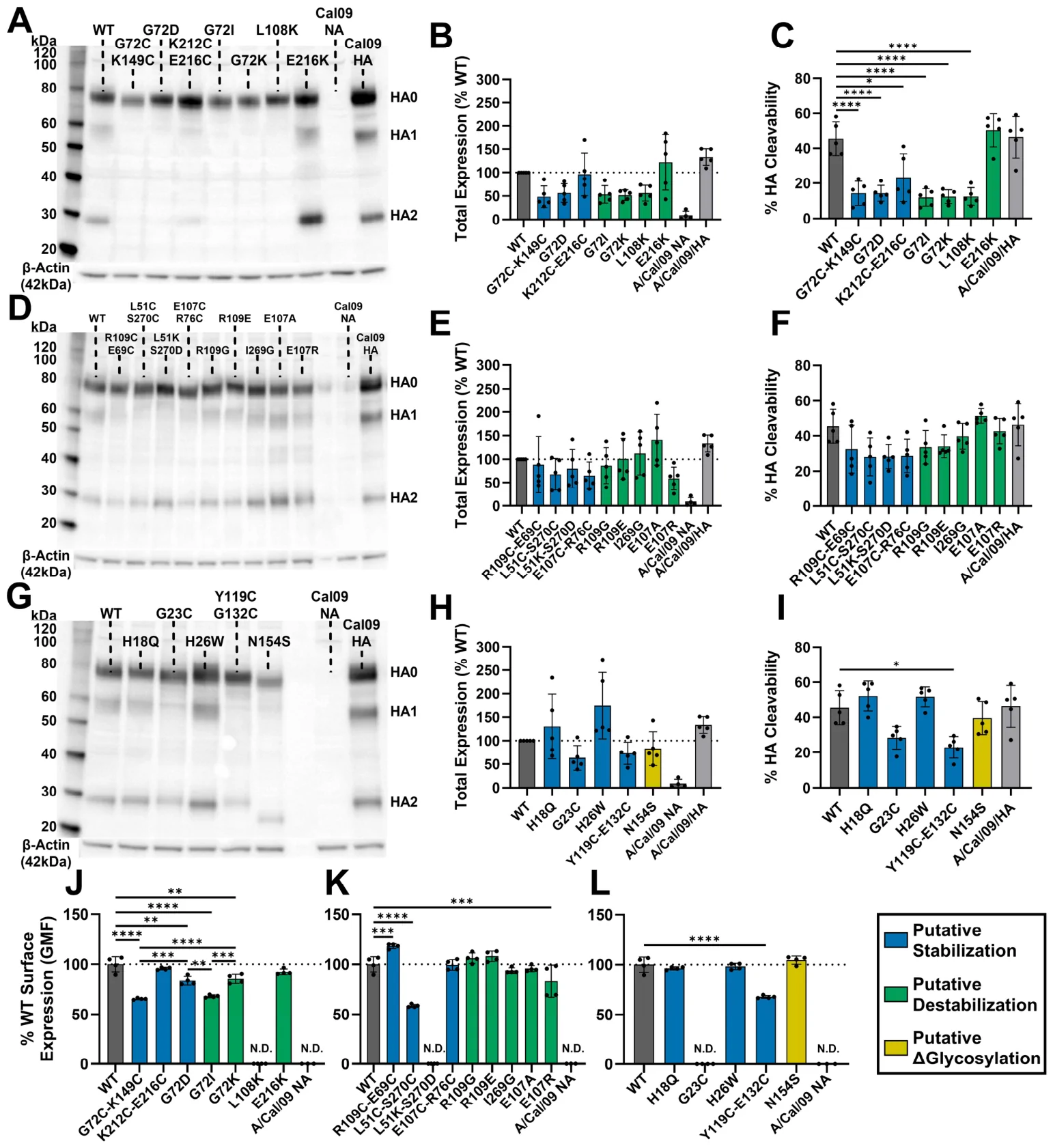
In vitro expression and cleavage of HA constructs. HEK-293T cells were transfected with pCAGGS expression plasmids encoding WT and mutant HAs, as well as Cal/09 HA and NA, then incubated for 24 h before TPCK-trypsin treatment and subsequent lysis. Data are normalized to the WT (dotted line) and are shown as the mean ± SD. (**A**–**I**) Total cellular expression by Western blot analysis. The mean gray values of bands from five independent experiments were analyzed with ImageJ. All gels within a biological replicate were run in parallel. Data are shown for (**A**–**C**) head substitutions, (**D**–**F**) head–stalk substitutions, and (**G**–**I**) stalk substitutions. (**A**,**D**,**G**) Representative blots. (**B**,**E**,**H**) Total cell expression (HA0 + HA1 + HA2). (**C**,**F**,**I**) HA activation as the percentage of cleaved HA [(HA1 + HA2)/(HA0 + HA1 + HA2) × 100]. (**J**–**L**) Cell-surface expression measured by flow cytometry and staining with the mAb CR6261. Data are from four experimental repeats, with *n* = 5000 HA-positive singlets per repeat. N.D indicates not detected. The bar charts show data for (**J**) head substitutions, (**K**) head–stalk substitutions, and (**L**) stalk substitutions. Data were compared by one-way ANOVA with Tukey’s multiple comparisons correction. * *p* < 0.05, ** *p* < 0.01, *** *p* < 0.001, **** *p* < 0.0001.

Western blot analysis was used to measure the total HA cellular expression and the percentage of HA cleaved by exogenous trypsin treatment for each construct relative to WT (Figure 2A–I; Table 2). Differences in signal intensity were visible between some HA banding patterns (Figure 2A,D,G), but these were not statistically significant (Figure 2B,E,H). G72C-K149C, G72D, K212C-E216C, G72I, G72K, and L108K exhibited reduced trypsin cleavage (*p* < 0.0001, *p* < 0.0001, *p* = 0.0323, *p* < 0.0001, *p* < 0.0001, and *p* < 0.0001, respectively), and E216K trypsin cleavability was maintained (Figure 2C). No modification located at the structural interfaces between the head and stalk domains resulted in a significant decrease in total cellular expression or trypsin cleavage. In the stalk, G23C HA cleavage was reduced, but the change was not statistically significant. However, Y119C-E132C exhibited a significant reduction (*p* = 0.0282) (Figure 2I). N154S was selected for vaccine formulation because the migration of its HA2 was consistent with its expected glycosylation deletion (Figure 2G) [46]. No substitutions were excluded based on total cell expression alone.

Next, cell-surface expression was assessed by flow cytometry (Figure 2J–L and Figure S1; Table 2). This method used binding of the CR6261 antibody to an epitope that targets an epitope which does not overlap with the test-set substitutions [66]. L108K, L51K-S270D, and G23C showed no cell-surface expression (Figure 2J–L; Table 2), whereas cell-surface expression of G72C-K149C, G72I, L51C-S270C, and Y119C-E132C was less than 70% of that of WT (*p* < 0.0001). Surface expression of G72D, G72K, and E107R, although reduced, was greater than 80% of that of WT (*p* = 0.0013, *p* = 0.0087, and *p* = 0.0007, respectively). The expression of G72D and G72K was greater than that of G72C-K149C (*p* = 0.0002, *p* < 0.0001) and G72I (*p* = 0.0021, *p* = 0.0004), respectively. R109C-E69C expression was 118% of WT expression (*p* = 0.0002) (Figure 2K; Table 2). G72C-K149C, G72I, L108K, L51C-S270C, L51K-S270D, G23C, and Y119C-E132C were excluded from further study because of the reductions in HA cell-surface expression.

### 3.3. In Vitro Stability of Mutant HAs

**Figure 3:**
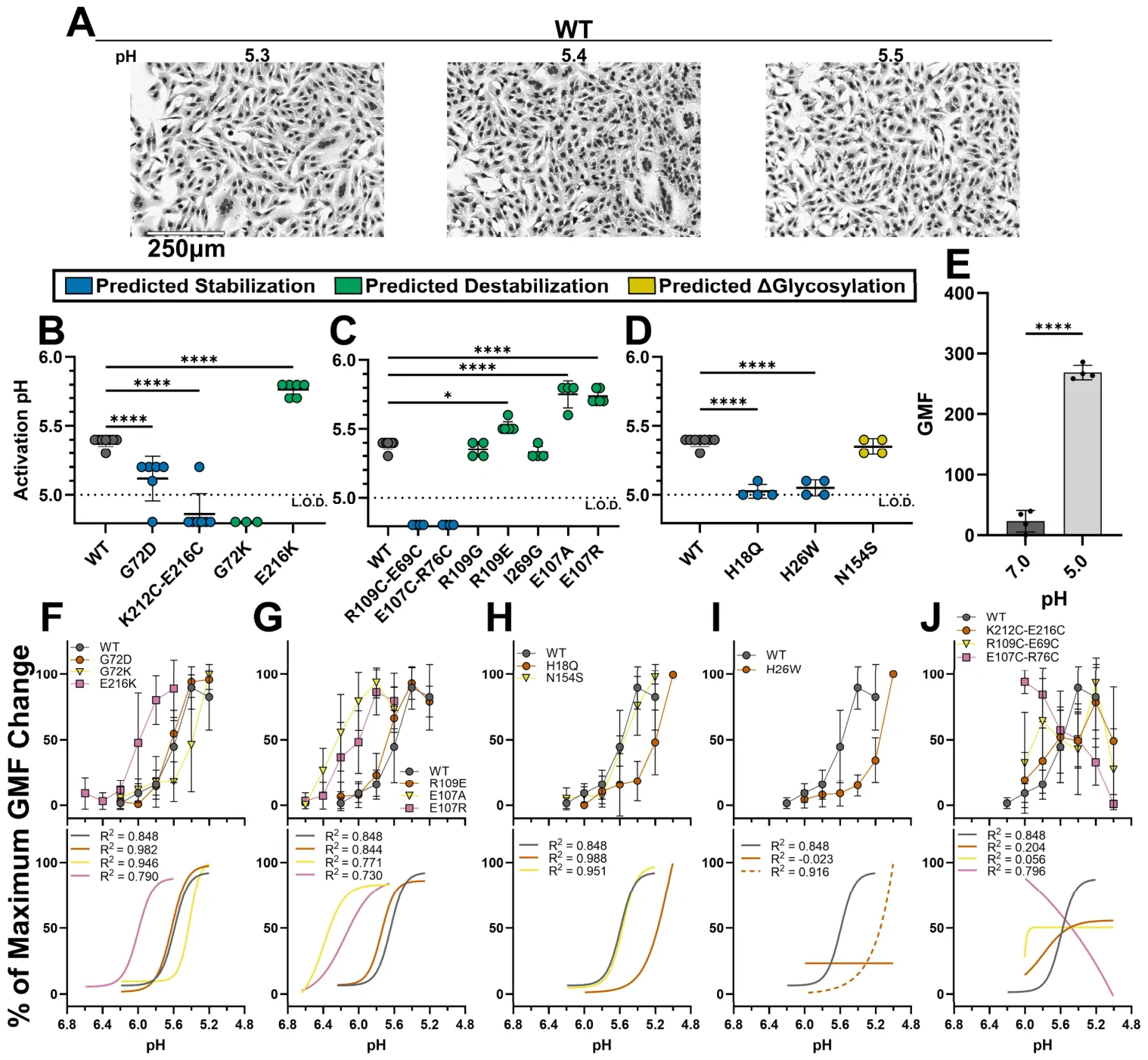
Stability of pCAGGS-expressed HA proteins. Samples within a given experiment were analyzed in parallel. (**A**–**D**) HA functional stability analyzed by a syncytia formation assay within the pH range of 6.0–5.0. The activation pH is defined as the highest pH for syncytia formation. Data represent *n* ≥ 3 independent experiments and are shown as the mean ± SD. Experiments without syncytia formation are denoted as below the limit of detection (LOD, dotted line). (**A**) Representative micrographs showing syncytia formation in cells transfected with WT HA at different pHs. (**B**–**D**) Activation pH data for (**B**) head substitutions, (**C**) head–stalk substitutions, and (**D**) stalk substitutions. Significance was assessed by one-way ANOVA with Dunnett’s multiple comparisons correction; * *p* < 0.05, **** *p* < 0.0001. (**E**–**J**) FluA-20 mAb binding (GMF) measured by flow cytometry. (**E**) Binding to WT HA at pH 7.0 and 5.0, assessed by Welch’s unpaired *t*-test. (**F**–**J**) Binding to non-excluded HAs within the pH range of 6.6–5.0. The percentage of the total fluorescence change, defined as maximum GMF − minimum GMF, is shown as a function of pH. The pH of conformation change is the pH for 50% fluorescence change, calculated by nonlinear least-squares regression to a symmetric four-parameter dose–response curve. Data represent *n* ≥ 3 repeat experiments and are shown as the mean ± SD. The graphs show data for (**F**) head substitutions, (**G**) head–stalk substitutions, (**H**) stalk substitutions, (**I**) H26W, as fitted to dose–response (solid) and exponential growth (dashed) curves, and (**J**) disulfide substitutions.

The HA head and stalk domains both undergo structural changes during fusogenic refolding, and substitutions in both influence HA conformational stability [67]. We screened the HA mutants for functional stability, measuring syncytia formation in HA-transfected cells pulsed between pH 5.0 and pH 6.0 (Figure 3A and Figure S2; Table 2). The highest pH at which syncytia formed was termed the HA activation pH, and this value was measured for all mutants (Figure 3B–D and Figure S2). HAs with substitutions that were robustly expressed were excluded from further analysis if their activation pH was similar to that of WT HA (*p* ≥ 0.05) (Figure 3B–D; Table 2). G72K, K212C-E216C, R109C-E69C, and E107C-R76C did not form syncytia reliably. G72D, H18Q, and H26W had reduced activation pH values (*p* < 0.0001), suggestive of enhanced stability (Figure 3B–D and Figure S2B–F; Table 2). Interestingly, syncytia formation by G72D was observed less frequently than with the WT HA but was still visible (Figure S2B); this is consistent with reduced trypsin activation (Figure 2C), as well as slight reductions in cell-surface expression (Figure 2J). R109G and I269G had activation pH values similar to that of the WT HA. N154S glycosylation ablation did not alter the activation pH. E216K, R109E, E107A, and E107R had increased activation pH values consistent with HA destabilization (*p* < 0.0001, *p* = 0.0183, *p* < 0.0001, and *p* < 0.0001, respectively). None of the expression-defective HA modifications excluded from further study experienced syncytia formation (Figure S2). While this may be due to alterations in stability, it is likely that the relative lack of cell-surface expression serves as a barrier to syncytia formation, though more work is needed to conclusively determine this. Nevertheless, G72D exhibited depressed cell-surface expression compared to WT, but also formed syncytia at a resolvable activation pH. This suggests that expression of all candidates which passed cell-surface expression screening was of sufficient quantities to support syncytia formation, given the comparable expression of G72K to G72D.

Overall, 12 mutant HA candidates were advanced for study in mRNA-LNPs. All exhibited robust in vitro expression. Eleven exhibited distinct functional stability phenotypes in vitro, whereas N154S putatively ablated an HA2 glycosylation site.

HA head domain conformation was then examined to better understand the region-specific stability of the advancing substitutions. This was done by measuring the pH-dependent binding properties of the FluA-20 mAb, which binds an epitope between conformationally dynamic RBDs [9,29]. Flow cytometry revealed that FluA-20 binding to HA treated at low pH was significantly greater than binding to HA at neutral pH (*p* < 0.0001), indicating its specificity to low-pH conformations (Figure 3E and Figure S3A). Binding, including the pH of conformational change (the pH for a 50% binding change), was then measured against pH for all expressing HA mutants (Figure 3F–J and Figure S3B–D; Table 2). For the advancing mutants, the pH of conformation change was similar to that of the WT for G72D, elevated for E216K, E107A, and E107R, and reduced for G72K and H18Q (Figure 3F–H; Table 2). Although destabilized, R109E exhibited WT-like head stability reflective of its modest phenotype. FluA-20 binding to N154S was also similar to its binding to the WT. H26W exhibited exponential binding at the lowest pHs tested, suggestive of enhanced head stability (Figure 3I). FluA-20 binding to the K212C-E216C and R109C-E69C designed disulfides was not strongly connected to pH. Uniquely, FluA-20 binding to E107C-R76C was directly proportional to pH (Figure 3J).

### 3.4. HA Mutant mRNA-LNP Vaccine Immunogenicity

**Figure 4:**
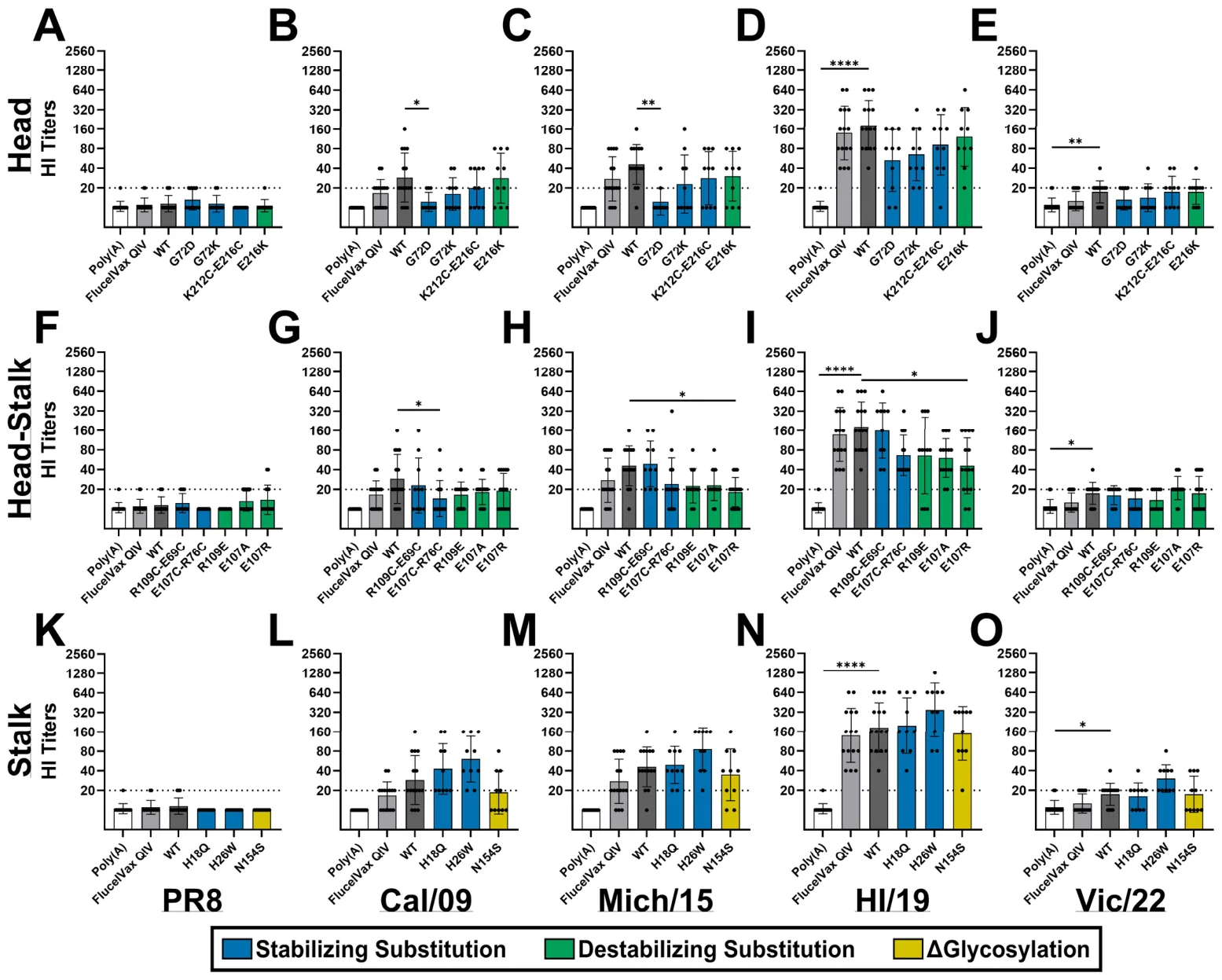
Hemagglutination inhibition (HI) activity of day 28 mutant HA mRNA-LNP post-vaccination sera. Groups of mice (*n* = 10–15 per treatment group) were injected intramuscularly with poly(A) mRNA-LNP (1 µg) or Flucelvax QIV Fall 2020–2021 (1.5 µg pH1N1 HA). Mice in the WT (HI/19 HA) and mutant candidate mRNA-LNP groups were given 0.5 µg each. Reciprocal HI titers are shown as the geometric mean ± SD. The limit of detection (dotted line) was 20. HI titers are shown for: (**A**–**E**) head mutants, (**F**–**J**) head–stalk mutants, and (**K**–**O**) stalk mutants. HI titers were determined for an H1N1 viral panel comprising: (**A**,**F**,**K**) PR8, (**B**,**G**,**L**) Cal/09, (**C**,**H**,**M**) Mich/15, (**D**,**I**,**N**) HI/19, and (**E**,**J**,**O**) Vic/22. Significance was assessed with a Kruskal–Wallis test and Dunn’s multiple comparisons correction. * *p* < 0.05, ** *p* < 0.01, **** *p* < 0.0001.

To study the immunogenic impact of these substitutions in vaccines, the panel of modified HA mRNA-LNPs was produced and characterized (Table S1). Next, groups of 10–15 mice were vaccinated with poly(A), Flucelvax QIV, WT, or mutant HA mRNA-LNPs. Hemagglutination inhibition (HI) titers against an H1N1 viral panel were then obtained for sera collected at 28 days post vaccination. Most HI titers after vaccination with head mutants were not significantly different from those obtained with the WT vaccine against any antigen (Figure 4A–E). However, G72D vaccination resulted in significantly reduced HI titers against Cal/09 and Mich/15 (*p* = 0.0291 and *p* = 0.0012, respectively) but conferred seroprotection against homologous HI/19 (HI titers ≥ 1:40), despite a 4-fold reduction compared to WT, albeit one that was not statistically significant (*p* = 0.1179). Differences between HI titers with WT and head–stalk mutant vaccines were largely non-significant (Figure 4F–J). E107C-R76C titers against Cal/09 were slightly, but noticeably, reduced (*p* = 0.0397). Minor, non-significant reductions in titers against homologous HI/19 antigens were seen with E107C-R76C, R109E, and E107A. However, with E107R, there were significant reductions in titers against Mich/15 and HI/19 (*p* = 0.0116 and *p* = 0.0120, respectively), despite the titers against the latter being seroprotective (Figure 4H,I). Stalk-mutant vaccine responses were comparable to those for the WT vaccine (Figure 4K–O).

**Figure 5:**
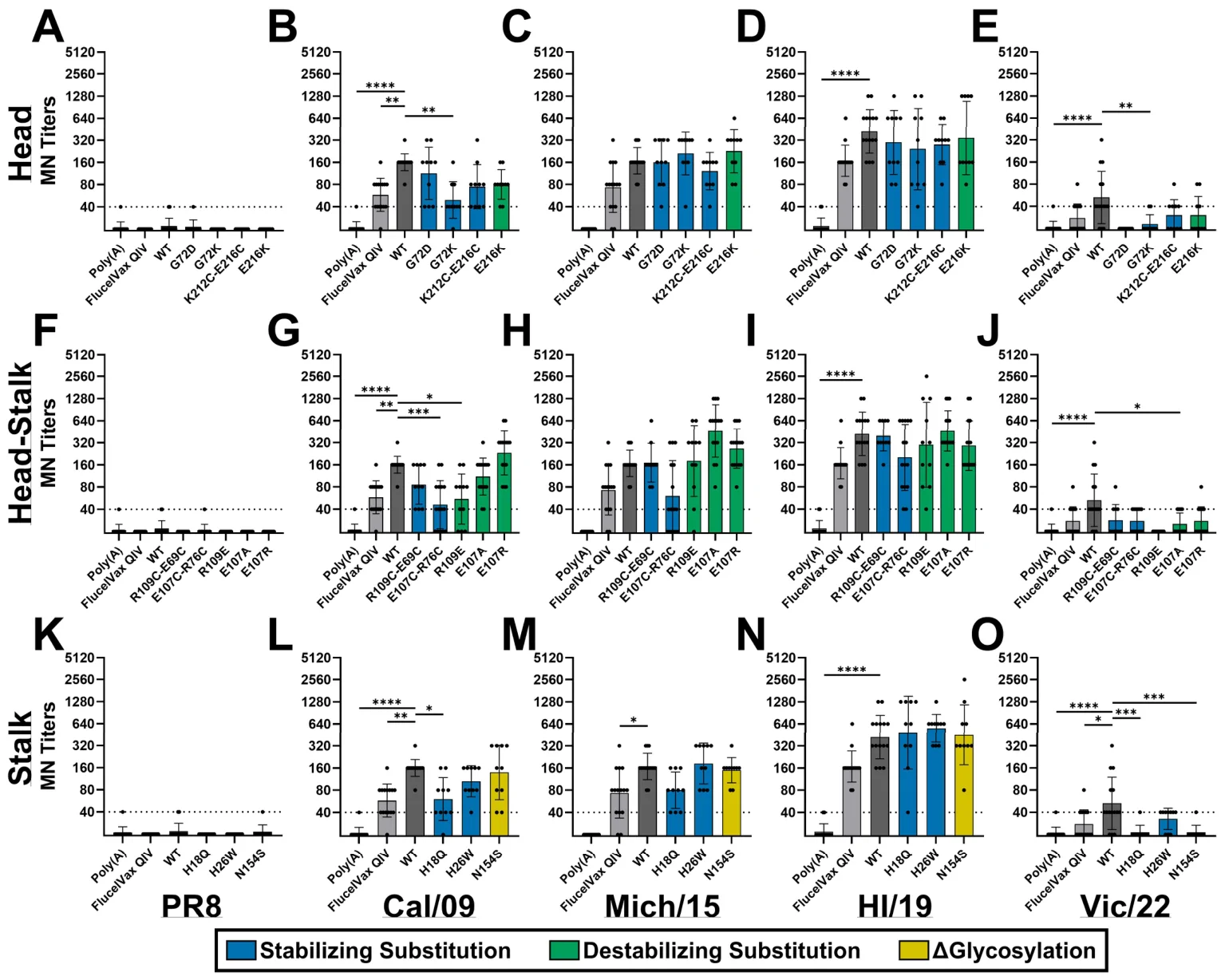
Microneutralization (MN) titers of day 28 mutant HA mRNA-LNP post-vaccination sera. Groups of mice (*n* = 10–15) were immunized with poly(A) mRNA-LNP (1 µg) or Flucelvax QIV Fall 2020–2021 (1.5 µg of pH1N1 HA). Mice in the WT (HI/19 HA) and mutant candidate mRNA-LNP groups were given 0.5 µg each. Serum samples were serially diluted 2-fold in technical triplicate. Data are represented as the geometric mean ± SD, and the limit of detection was a 1:40 serum dilution (dotted line). MN titers are shown for: (**A**–**E**) head mutants, (**F**–**J**) head–stalk mutants, and (**K**–**O**) stalk mutants. Titers were determined using an H1N1 virus panel comprising: (**A**,**F**,**K**) PR8, (**B**,**G**,**L**) Cal/09, (**C**,**H**,**M**) Mich/15, (**D**,**I**,**N**) HI/19, and (**E**,**J**,**O**) Vic/22. Significance was assessed with a Kruskal–Wallis test and Dunn’s multiple comparisons correction. * *p* < 0.05, ** *p* < 0.01, *** *p* < 0.001, **** *p* < 0.0001.

Day 28 post-vaccination sera microneutralization (MN) titers were also determined using the H1N1 viral panel. MN titers for head-mutant vaccinated mice were mostly comparable to those for WT-vaccinated mice, although G72K-elicited responses against Cal/09 were reduced (*p* = 0.0016) (Figure 5A–E). G72D titers against Vic/22 were non-detectable. This reduction was also seen in G72K-elicited responses against Vic/22 (*p* = 0.0055). MN titers with a head–stalk mutant vaccine were largely similar to those for WT-vaccinated mice (Figure 5F–J), although responses against Cal/09 with R109C-E69C and R109E mutant vaccines were reduced (*p* = 0.0005 and *p* = 0.0109, respectively). Mice vaccinated with E107A also exhibited a decreased response against Vic/22 (*p* = 0.0127). Titers elicited with stalk-mutant vaccines were similar to those obtained with WT vaccination, except for a slight decrease in H18Q titers against Cal/09 (*p* = 0.0201) (Figure 5K–O).

**Figure 6:**
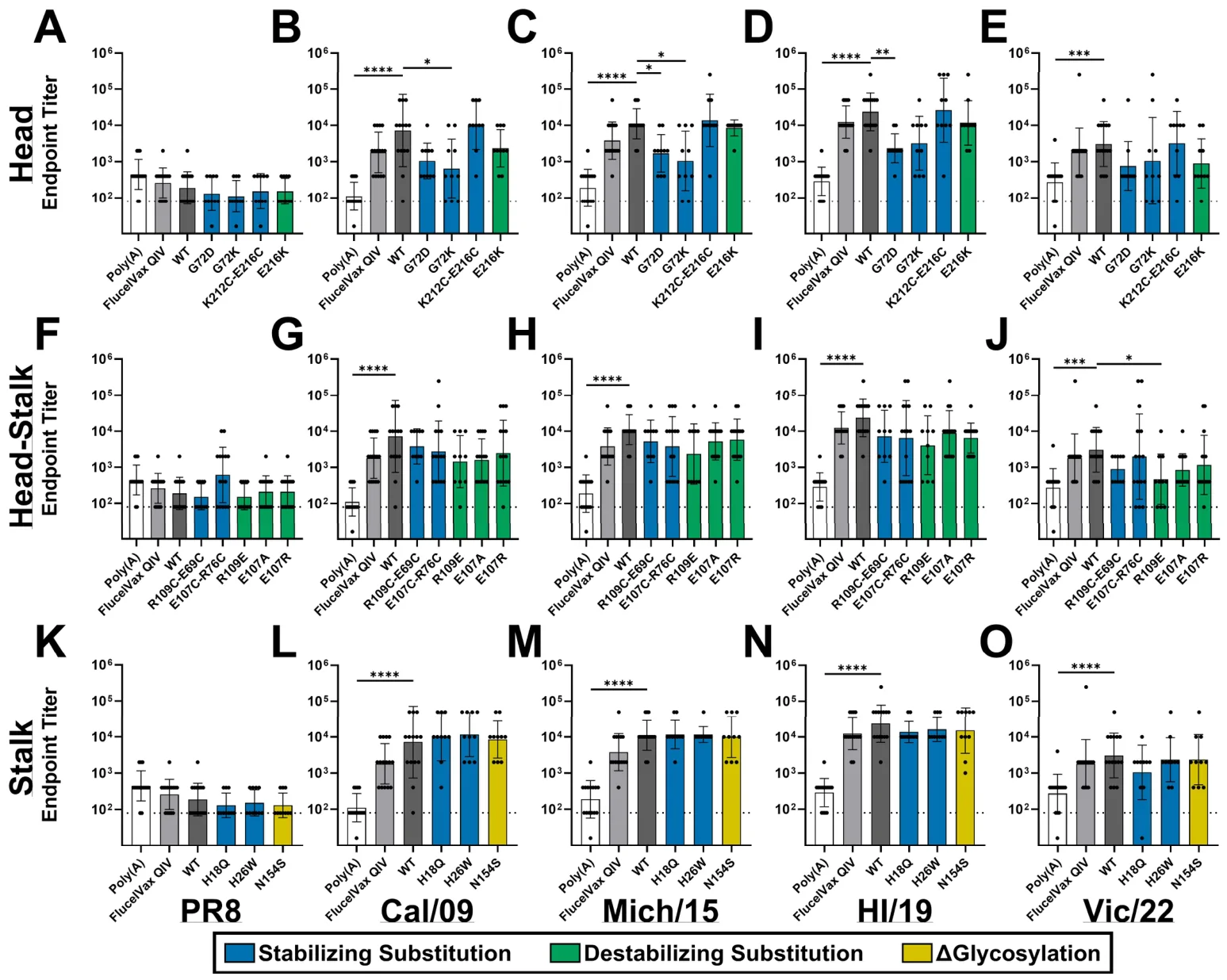
Total IgG ELISA titers of day 28 mutant-HA mRNA-LNP post-vaccination sera. Groups of mice (*n* = 10–15 total) were immunized with poly(A) mRNA-LNP (1 µg) or Flucelvax QIV Fall 2020–2021 (1.5 µg per HA). Mice in the WT (HI/19 HA) and mutant candidate mRNA-LNP groups were given 0.5 µg each. Serum samples were serially diluted 5-fold, with the limit of detection being the 1:80 dilution factor (dotted line), and total IgG titers were determined against an inactivated H1N1 whole-virus panel. Data are represented as the geometric mean ± SD. Data are shown for: (**A**–**E**) head mutants, (**F**–**J**) head–stalk mutants, and (**K**–**O**) stalk mutants. Titers were determined using an H1N1 virus panel comprising: (**A**,**F**,**K**) PR8, (**B**,**G**,**L**) Cal/09, (**C**,**H**,**M**) Mich/15, (**D**,**I**,**N**) HI/19, and (**E**,**J**,**O**) Vic/22. Significance was assessed with a Kruskal–Wallis test and Dunn’s multiple comparisons correction; * *p* < 0.05, ** *p* < 0.01, *** *p* < 0.001, **** *p* < 0.0001.

Day 28 post-vaccination sera were also analyzed by inactivated, whole-virus IgG ELISA (Figure 6). IgG titers with K212C-E216C and E216K vaccination were comparable to those with WT vaccination against all tested antigens (Figure 6A–E). However, vaccination with G72D resulted in reduced titers against all antigens except for PR8, although only the reductions in the responses against Mich/15 and HI/19 reached statistical significance (*p* = 0.0283 and *p* = 0.0079, respectively). G72K behaved similarly to G72D, with titer reductions against Cal/09 and Mich/15 reaching significance (*p* = 0.0456 and *p* = 0.0102, respectively). Head–stalk mutant IgG responses were also similar to those obtained post WT vaccination, except for R109E-elicited responses against Vic/22, which were reduced (*p* = 0.0228) (Figure 6F–J). Titers with stalk mutant vaccination were comparable to those with WT vaccination (Figure 6K–O).

The strongest serum responses were against homologous HI/19, whereas responses against Cal/09 and Mich/15 were reduced but observable (Figure 4, Figure 5 and Figure 6). Responses were even weaker against PR8 and Vic/22, for which the total IgG ELISA titers, as well as the HI and MN titers, were reduced to near or below the threshold of detection.

### 3.5. Mutant mRNA-LNP Weight Loss and Survival Against a Drifted pH1N1 Challenge

**Figure 7:**
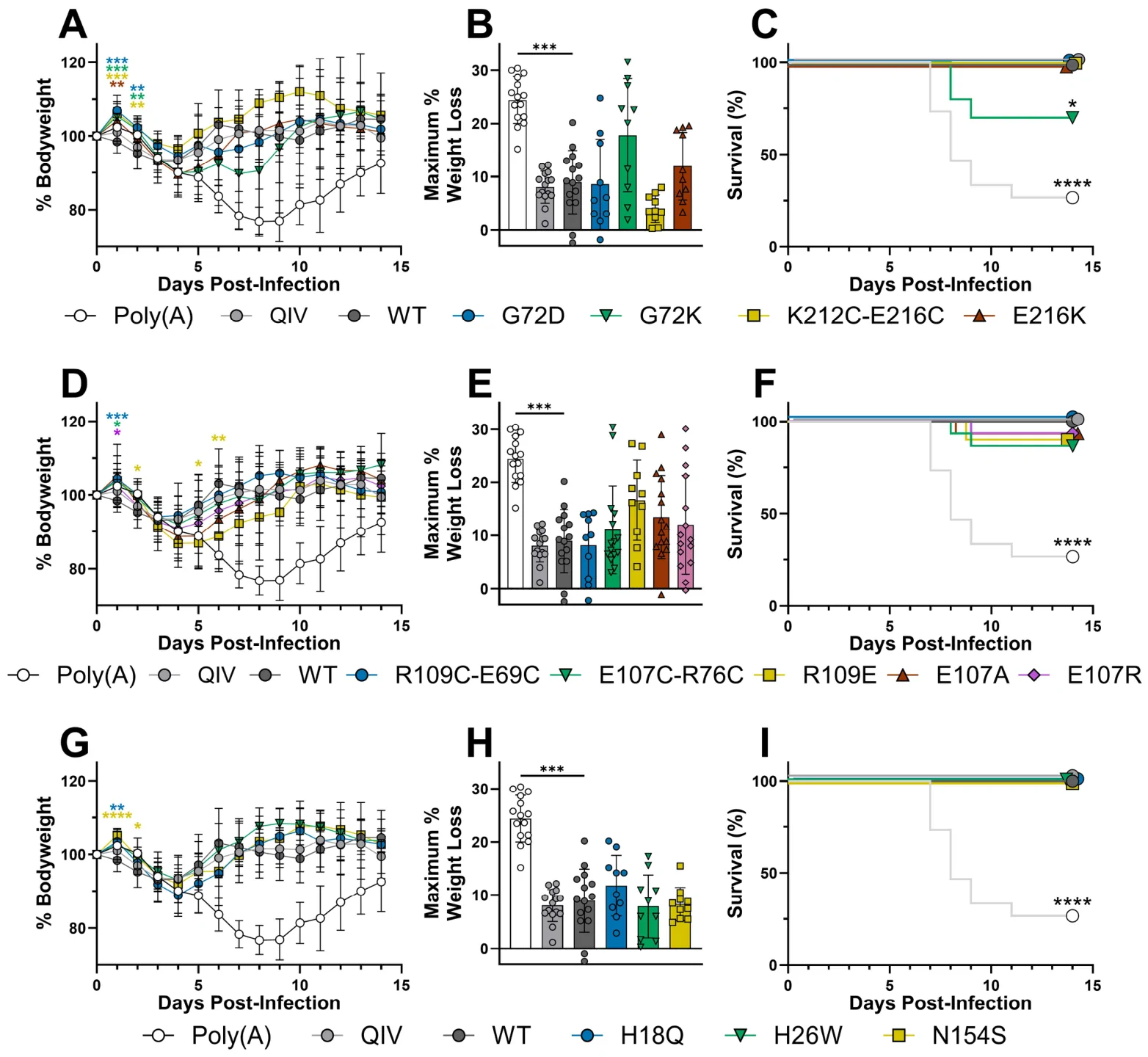
Weight loss and survival of mutant HA mRNA-LNP-vaccinated mice challenged with a drifted pH1N1 virus. Groups of mice were immunized with poly(A) mRNA-LNP (1 µg), Flucelvax QIV Fall 2020–2021 (1.5 µg of pH1N1 HA), WT (0.5 µg of HI/19 HA), or mutant (0.5 µg) mRNA-LNP vaccine per mouse. Mice were then challenged by intranasal inoculation with 10 × mLD_50_ of A/Victoria/4897/2022 (H1N1) at day 29 post vaccination. Weight loss and survival of the *n* = 10–15 total mice per treatment group were monitored for 14 days post challenge in parallel experiments. Data are shown for: (**A**–**C**) head mutants, (**D**–**F**) head–stalk mutants, and (**G**–**I**) stalk mutants. (**A**,**D**,**G**) Weight loss curves (mean ± SD) are compared to those for WT-vaccinated mice through day 7 post infection by matched mixed-effects analysis with a Geisser–Greenhouse variability correction and Dunnett’s multiple comparisons correction. (**B**,**E**,**H**) Maximum weight loss per mouse (mean ± SD) was compared to that for WT-vaccinated mice with a Kruskal–Wallis test with Dunn’s multiple comparisons correction. (**C**,**F**,**I**) Survival curves were compared to those for WT-vaccinated mice by log-rank tests. For all statistics, * *p* < 0.05, ** *p* < 0.01, *** *p* < 0.001, **** *p* < 0.0001.

To test the impact of the HA modifications on vaccine protection against a pH1N1 virus with significant antigenic drift from HI/19, mice were lethally challenged with A/Victoria/4897/2022 (Vic/22) 29 days after vaccination [68,69]. Weight loss and survival were monitored for 14 days post challenge (Figure 7). Poly(A)-mRNA-LNP-vaccinated mice experienced greater maximum weight loss (*p* ≤ 0.0001) than those vaccinated with WT, and greater weight loss between days 5 and 7 (*p* < 0.05), after which comparisons ceased as the animals in these groups died. Survival with the poly(A) vaccination was 27%, compared to 100% with WT vaccination (*p* ≤ 0.0001). Some differences in weight loss were seen on days 1 and 2 post-challenge between specific groups and WT, though these were minor (Figure 7A,D,G). Notably, R109E-vaccinated mice gained 10% and 14% less weight than WT-vaccinated mice on days 5 and 6 post infection (*p* = 0.0324 and *p* = 0.0092, respectively), suggestive of mildly impaired recovery (Figure 7D). Although not statistically different than the result for WT-vaccinated mice, the maximum weight loss experienced by mice vaccinated with head mutant and head–stalk mutant vaccines was variable (Figure 7B,E,H). Specifically, G72K vaccination resulted in an increase in maximum weight loss, which, although not statistically significant, coincided with a significantly decreased survival rate of 70% (*p* = 0.0254) (Figure 7B,C). This was in contrast to the 100% survival of G72D-vaccinated mice. Both maximum weight loss and survival of mice vaccinated with a head–stalk mutant were not statistically different from the corresponding values for WT-vaccinated mice, with R109C-E69C, E107C-R76C, R109E, E107A, and E107R vaccination resulting in 100%, 87%, 90%, 93%, and 93% survival, respectively (Figure 7E,F). Stalk mutant-vaccinated mice exhibited per diem weight loss, maximum weight loss, and survival comparable to that of WT-vaccinated mice (Figure 7H,I). These data suggest that the protection imparted by most of these mutant vaccine candidates against an antigenically drifted challenge virus is similar to that conferred by the WT.

### 3.6. Effects of E107 Modifications on Domain-Specific Antibody Responses and ADCC Activity

**Figure 8:**
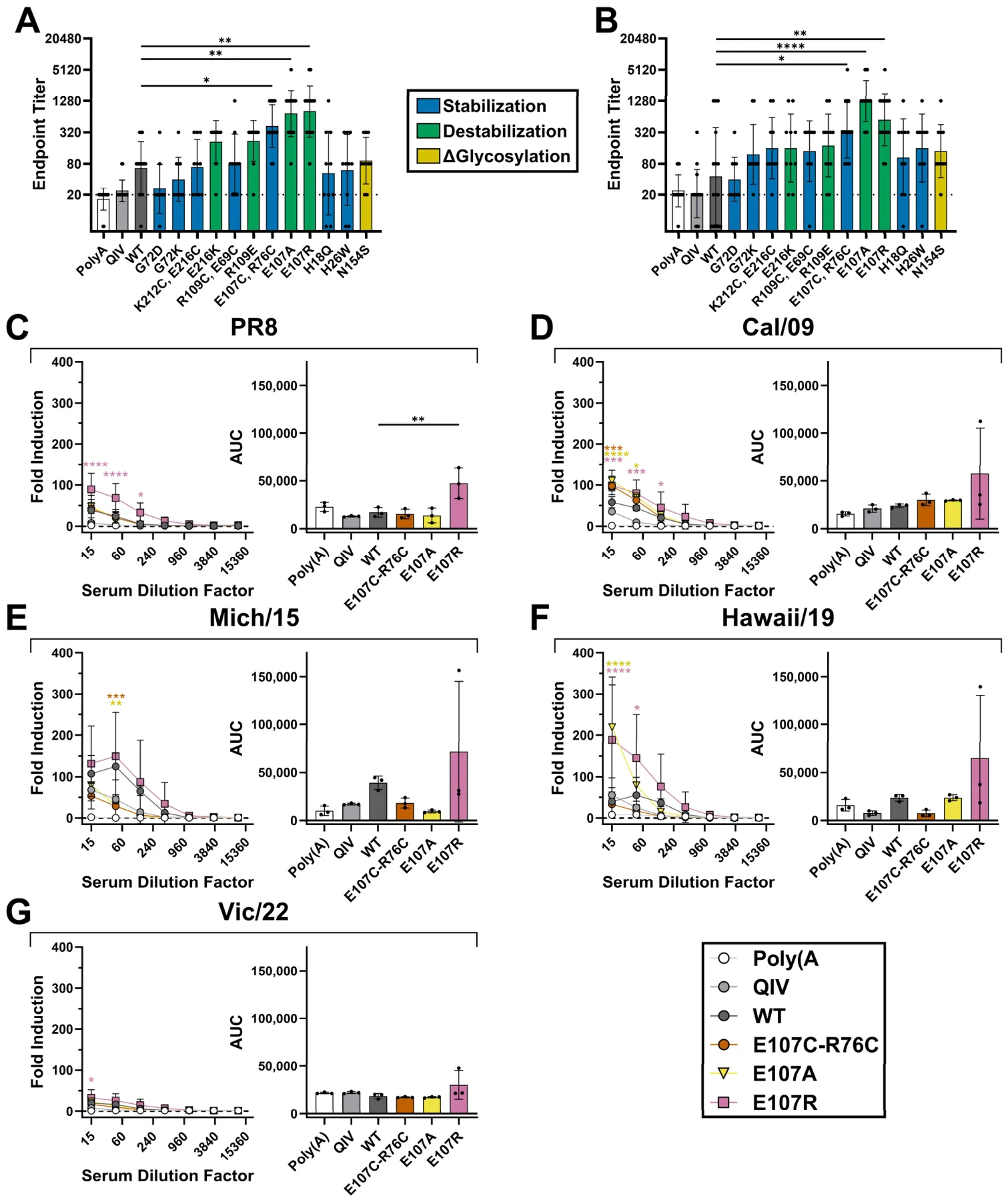
E107 mutant mRNA-LNP anti-HA stalk responses and ADCC activities. Mice (*n* = 10–15) were immunized with poly(A) mRNA-LNP, Flucelvax QIV, WT mRNA-LNP, or mutant mRNA-LNP, using 1 µg and 1.5 µg of pH1N1 HA, and 0.5 µg and 0.5 µg per mouse, respectively. (**A**,**B**) Stalk-directed IgG ELISA endpoint titers. Day 28 post-vaccination sera were serially diluted 4-fold; the threshold of detection was 1:20 (dotted line). Geometric means ± SDs are shown, with data compared to those for WT vaccination with a Kruskal–Wallis test and Dunn’s multiple comparisons correction. (**A**) Titers against a cH6/H1 chimeric HA [56]. (**B**) Titers against MiniHA #4900 stalk-only antigen [15]. (**C**–**G**) ADCC activity against H1N1 viruses as measured by reporter assays. Pooled day 28 post-vaccination sera from three different groups (*n* = 5 mice per group) were serially diluted 3-fold, with a threshold of detection of 1:15, then mixed with infected MDCK cells in technical triplicate. ADCC reporter cells were added and the mixture incubated for 6 h at 37 °C. Data were collected as luminescence in relative light units (RLUs). Results are shown as means ± SDs, represented both as fold-induction curves (RLU_sample_ − RLU_background_)/(RLU_no-sera controls_ − RLU_background_) versus sera dilution and as the areas under these curves (AUCs). ADCC activity data are shown for: (**C**) PR8, (**D**) Cal/09, (**E**) Mich/15, (**F**) Hawaii/19, and (**G**) Vic/22. ADCC data were compared to those for WT vaccination by two-way ANOVA with Dunnett’s multiple comparisons correction. * *p* < 0.05, ** *p* < 0.01, *** *p* < 0.001, **** *p* < 0.0001.

We next examined the HA stalk-specific antibody responses of mutant-vaccinated mice, as the stalk domain is relatively conserved antigenically and is widely targeted in next-generation influenza vaccine development [6]. IgG ELISAs were conducted against chimeric HAs consisting of foreign H6 heads and H1 stalks (Figure 8A) [56]. Vaccination with E107C-R76C, E107A, and E107R yielded 6-fold, 11-fold, and 12-fold higher endpoint titers, respectively, than vaccination with WT (*p* = 0.0255, *p* = 0.0016, and *p* = 0.0013, respectively). IgG responses against an H1 stalk-only antigen (MiniHA #4900) [15] showed an 8-fold, 28-fold, and 12-fold increase in endpoint titers with, respectively, E107C-R76C, E107A, and E107R vaccination when compared to titers with WT vaccination (*p* = 0.0313, *p* < 0.0001, and *p* = 0.0019, respectively) (Figure 8B). Given the similarities between the ELISA antigens, these results indicate that E107C-R76C, E107A, and E107R enhance H1 stalk-domain responses.

As anti-stalk antibodies protect through an Fc-mediated effector function [70], we measured the ADCC activity of E107 mutant sera against H1N1 viruses by using a reporter assay (Figure 8C–G). Sera from E107R-vaccinated mice displayed 2-, 3-, and 10-fold increases in fold-induction against PR8 at 1:15, 1:45, and 1:135 dilutions, respectively (*p* < 0.0001, *p* < 0.0001, and *p* = 0.0195, respectively), as well as greater AUCs when compared with sera from other groups (*p* = 0.0040) (Figure 8C). E107R fold-induction against Cal/09 was also elevated at 1:15, 1:45, and 1:135 dilutions (*p* = 0.0001, *p* = 0.008, and *p* = 0.0326, respectively) (Figure 8D). Similarly, E107A induced increases against Cal/09 at 1:15 and 1:45 dilutions (*p* < 0.0001 and *p* = 0.0113, respectively). E107C-R76C also induced increased activity against Cal/09 at the 1:15 dilution (*p* = 0.0002). E107C-R76C and E107A induced significantly decreased activity against Mich/15 at the 1:45 dilution (*p* = 0.0025 and *p* = 0.0034, respectively) and non-significant reductions at the 1:135 dilution (*p* = 0.0868 and *p* = 0.0727, respectively) (Figure 8E). However, E107R fold-induction was similar to that of WT. E107A and E107R exhibited greater fold-induction against Hawaii/19 than WT at the 1:15 serum dilution (*p* < 0.0001), and E107R showed enhanced induction at the 1:45 dilution (*p* = 0.0137) (Figure 8F). There was a modest increase in E107R fold-induction against Vic/22 at the 1:15 dilution (*p* = 0.0139) (Figure 8G). Although there were significant differences noted at specific dilutions against specific antigens, especially in E107R, all mutant sera AUCs were similar to those of WT against all antigens, except for E107R against PR8 (Figure 8D–G).

### 3.7. E107 Mutant mRNA-LNP Protection Against Lethal H1N1 Challenges

**Figure 9:**
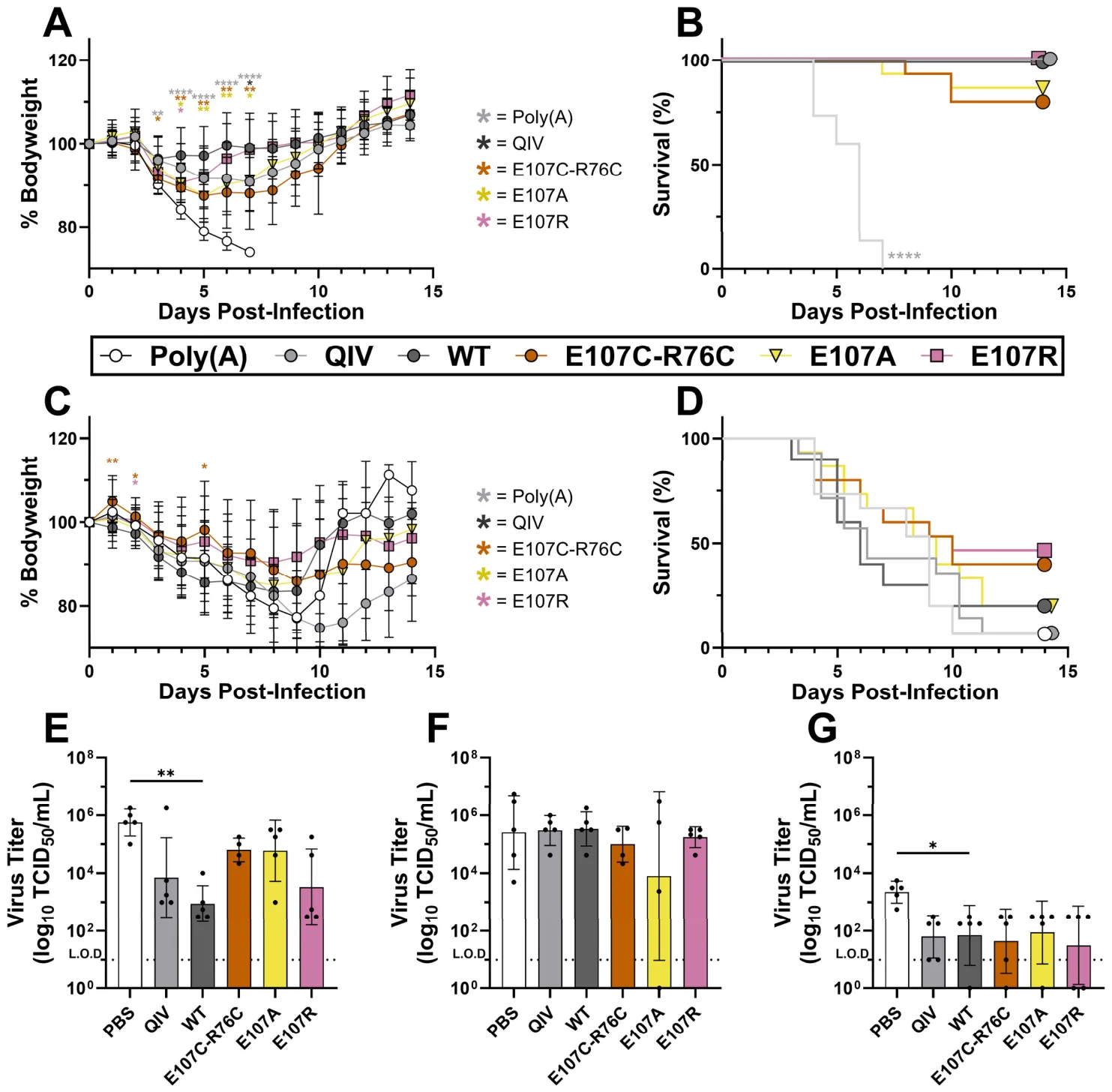
E107 mutant vaccine protection against H1N1 challenge. (**A**–**D**) DBA/2J mice (*n* = 10–15) were immunized with poly(A) mRNA-LNP, Flucelvax QIV Fall 2020–2021, WT, or mutant mRNA-LNP vaccines in parallel. Mice were then challenged by intranasal inoculation with 10 × mLD_50_ of H1N1 viruses. Weight loss per day (mean ± SD) and survival were then monitored for 14 days post infection. Weight loss was compared to that of WT-vaccinated mice by using a two-way ANOVA with Dunnett’s multiple comparisons correction. Survival curves were compared individually to those for WT-vaccinated mice by log-rank tests. (**A**,**B**) Weight loss and survival with MA-Cal/09 challenge. (**C**,**D**) Weight loss and survival with PR8 challenge. (**E**–**G**) In a separate experiment, groups of mice (*n* = 4–5) were immunized as above, using PBS^−/−^ (50 µL) instead of 1 µg of poly(A) mRNA-LNP as a negative control. At 3 days post challenge, lungs were harvested, homogenized, and frozen at −80 °C, then used to determine the viral load as log_10_ TCID_50_/mL. 10^1^ TCID_50_/mL is limit of detection (dotted line). Data are shown as the geometric mean ± SD and were compared to those for WT-vaccinated mice by a Kruskal–Wallis test with Dunn’s multiple comparisons correction. Mice were challenged with: (**E**) 10 × mLD_50_ of MA-Cal/09 at 29 days post vaccination, (**F**) 10 × mLD_50_ of PR8 at 34 days post vaccination, or (**G**) 10 × mLD_50_ of Vic/22 at 34 days post vaccination. The significance of all comparisons is denoted as follows: * *p* < 0.05, ** *p* < 0.01, **** *p* < 0.0001.

To investigate the impact of the E107 mutants on mRNA-LNP breadth of protection, we challenged mice with either the distantly related pH1N1 virus MA-Cal/09 or the heterologous PR8 virus at 29 days post vaccination. Weight loss and survival were monitored for 14 days post challenge (Figure 9A–D). Weight loss in mutant-mRNA-LNP-vaccinated mice was compared to that in WT-mRNA-LNP-vaccinated mice through day 7 post MA-Cal/09 challenge. Poly(A) mRNA-LNP-vaccinated mice lost more weight than WT-vaccinated mice on day 3 post infection (*p* = 0.0027), a pattern that continued through days 4–7 (*p* < 0.0001) (Figure 9A). QIV-vaccinated mice lost 8% more weight than WT-vaccinated mice on day 7 post challenge (*p* = 0.0158). E107C-R76C-vaccinated mice lost more weight than WT-vaccinated mice between days 3 and 7 (*p* = 0.0320, *p* = 0.0070, *p* = 0.0017, *p* = 0.0028, and *p* = 0.0024, respectively), with the largest difference being 11%. Similar to E107C-R76C mice, E107A-vaccinated mice experienced greater weight loss between days 4–7 post challenge (*p* = 0.0118, *p* = 0.0009, *p* = 0.0051, and *p* = 0.0193, respectively), with the greatest difference being 10%. E107R-vaccinated mice lost 7% more weight than WT-vaccinated mice on day 4 post challenge (*p* = 0.0185). With the exception of poly(A)-vaccinated mice (*p* < 0.0001), survival in any group was not significantly different from the 100% survival of WT-vaccinated mice (Figure 9B). Weight loss in mutant-mRNA-LNP-vaccinated and WT-mRNA-LNP-vaccinated mice was compared for 6 days after lethal PR8 challenge (Figure 9C). Poly(A)-, QIV-, and E107A-vaccinated mice experienced weight loss comparable to that of WT-vaccinated mice. However, E107C-R76C-vaccinated mice showed slightly less weight loss on days 1 and 2 post challenge (*p* = 0.0014 and *p* = 0.0361, respectively), and 13% less weight loss on day 5 (*p* = 0.0291). E107R-vaccinated mice lost slightly less weight than WT-vaccinated mice on day 2 post challenge (*p* = 0.0357). E107C-R76C– and E107R-vaccinated mice exhibited 20% and 27% greater survival, respectively, than WT-vaccinated mice, although these differences were not significant (Figure 9D).

In a separate experiment, we examined the viral burden in the lungs of vaccinated mice at 3 days post challenge (Figure 9E–G). Mice vaccinated using the negative control (PBS) had higher viral burdens than mice given WT mRNA-LNPs after challenge with MA-Cal/09 (*p* = 0.0026) (Figure 9E). However, no significant difference was found between any group and WT-vaccinated mice in the response against PR8 (Figure 9F). Additional analysis post Vic/22 challenge found higher viral burdens in the lungs of PBS-vaccinated mice than mice given WT mRNA-LNPs (*p* = 0.0147) (Figure 9G). The lung viral burdens of E107 mutant-vaccinated mice did not significantly differ from those of WT-vaccinated mice challenged with these viruses.

Mice given E107 mutant mRNA-LNPs experienced greater weight loss than WT-vaccinated mice when challenged with MA-Cal/09, although this varied in magnitude depending on the substitution identity. However, differences in survival were not statistically significant, nor were lung viral burdens significantly greater than those in WT-vaccinated mice (Figure 9A,B,E). Weight loss, survival, and viral lung burden phenotypes after lethal PR8 or Vic/22 challenge were largely similar in mutant-vaccinated and WT-vaccinated mice, further indicating that the immunogenicities of E107 substitution mRNA-LNPs were largely comparable to that of the WT vaccine against these viruses (Figure 7D–F and Figure 9C,D,F,G).

## 4. Discussion

This study establishes a practical antigen-engineering pipeline to identify hemagglutinin substitutions that reshape the quality of mRNA-LNP vaccine-elicited immunity without broadly compromising protection. By combining an in vitro pCAGGS-based screen of H1 HA expression, maturation, surface display, and stability phenotypes with downstream mRNA-LNP vaccination and lethal challenge studies in mice, we identified E107 as a previously underappreciated site capable of altering HA domain immunogenicity. In contrast to most substitutions, which elicited WT-like serological responses and protection, E107C-R76C, E107A, and especially E107R increased antibody responses against conserved H1 stalk antigens while preserving strain-specific responses. These changes were accompanied by measurable increases in ADCC activity against multiple H1N1 antigens and, in some E107-vaccinated groups, numerical improvements in survival after heterologous PR8 challenge. Although additional structural and functional studies are needed to define the mechanism and determine whether these stalk-biased responses causally contribute to protection, these findings demonstrate that targeted HA substitutions can tune the specificity of vaccine-induced humoral immunity while maintaining protective efficacy. More broadly, this work highlights the utility of mutational antigen screens for discovering HA modifications that may improve next-generation influenza vaccines designed to elicit broader, stalk-directed antibody responses.

Reports have emphasized the importance of mRNA-LNP vaccine antigen expression in eliciting protection [59,71,72]. Indeed, both total antigen cellular expression and cell-surface expression have been linked with the magnitude of adaptive immunity [73,74]. The mild but noticeable enhancements in R109C-E69C cell-surface expression are notable in that they are consistent with improvements in soluble SARS-CoV-2 Spike-protein ectodomain expression upon the introduction of a stabilizing disulfide bond [75], though in that case, folding was thought to be less reliable than in WT. Similar expression benefits yielded by engineering disulfide bonds in HIV-1 glycoproteins and RSV F-proteins have been reported [76,77]. It is possible that R109C-E69C, as well as K212C-E216C and E107C-R76C, may enhance the expression of soluble HA ectodomains given the similarities (and benefits in R109C-E69C) in cell-surface expression relative to WT. If validated in soluble constructs, this information could prove useful in recombinant protein vaccine platforms. Further investigation of these modifications for their effect on the expression of soluble HA constructs is therefore warranted. In addition, the effects of the non-disulfide HA stabilizing mutations on soluble HA expression have not been explored here, and are also worth investigating. It was recently shown that stabilizing the histidine switch 1 region in H1 HA prolonged the presence of cell-surface expressed HA after transient modified-mRNA transfection [34]. While this was done in membrane-bound HA, the enhanced expression over time shown in these studies is worth investigating in soluble HA contexts.

In addition to expression, HA trypsin cleavage is required for H1 maturation into functional molecules capable of undergoing fusion-associated conformation changes [78]. Western blot analysis coupled with exogenous trypsin treatment revealed universal expression of HA precursors but substitution-specific HA cleavage phenotypes (Figure 2A–I). Given the limited exogenous trypsin cleavage and the significantly diminished cell-surface expression profiles of L51C-S270C, L51K-S270D, L108K, G23C, and Y119C-E132C, the data suggest that these residues may be important in HA folding and trafficking to the cell surface [79,80]. In contrast, G72-associated substitutions exhibited varying cell-surface expression phenotypes but universal obstruction of trypsin cleavage (Figure 2C,J). Such reductions were seen with both G72D and G72K, despite their cell-surface expression being comparable to that of E107R. Similarly, K212C-E216C HA cleavage was reduced despite unaltered cell-surface expression. Structural mechanisms may explain the reduced cleavage observed in these mutants, which is surprising given the large distance between these residues and the cleavage site [81]. Consistent with the migration patterns found in previous experiments with H3 HAs, trypsin treatment permitted the identification of the ablated N154S glycosylation site in HA2 (Figure 2G) [46]. Interestingly, HA cleavage has been documented to alter the binding of mAbs [29]. However, the data here support but cannot conclusively confirm that the role of trypsin cleavage in HA vaccine immunogenicity is minor, if present at all. G72D and G72K reduced HA cleavability and elicited similar serological immunogenicities that were noticeably diminished from WT, with G72K-vaccinated mice showing decreased survival post Vic/22 challenge. However, K212C-E216C also had reductions in trypsin cleavability, but elicited WT-like serology and protection from challenge. Consistent with these data, one study also found that ablating a pH1N1 HA cleavage site had no major effect on mRNA-LNP vaccine responses [32]. However, the relation between HA maturation and ability to undergo conformational changes is known, and could be important in the context of work showing that modifications which stabilize HA2 enhance influenza mRNA-LNP vaccine immunogenicity [33,34]. Additional studies of HA expression and cleavability in mRNA-LNP-vaccinated tissues would therefore be useful in determining the cleavage states and possible conformational ensembles of HA antigens in vivo.

Most substituted HAs with altered fusogenic stability exhibited pH-dependent conformational changes that were higher than the activation pH (Figure 3, Figures S2 and S3; Table 2). This is consistent with previous work [27] and probably reflects FluA-20 epitope exposure as the head domain detaches from the stalk early during fusion [82]. G72 mutant fusogenicities were stabilized in G72D and ablated in G72K, which may be indicative of indirect stabilizing effects, given their distance from the fusion machinery. K212C-E216C exhibited ablated fusogenicity and pH-independent FluA-20 binding indicative of inconsistent epitope accessibility, and this reflects K212C-E216C phenotypes seen in H3 [17,44]. In contrast, K216E showed greater instability both functionally and at the RBD–RBD interface, which is suggestive of repulsive forces acting here and reflects work on H5 HAs (Figure 3B,F) [42]. Uniquely, E107C-R76C ablated fusogenicity within the tested range but exhibited FluA-20 binding that was directly proportional to pH (Figure 3). This putative disulfide mutant could impose the adoption of a rigid, radially expanded RBD conformation that compresses at low pH because of a clash with refolding HA2. Structural studies are needed to explain these phenotypes precisely. Although recent studies have found relations between HA stability and mRNA-LNP vaccine antigenicity, the in vivo conformations of these antigens are unclear [33,34]. Lymphoid tissues are thought to be slightly alkaline [83], potentially predisposing HA antigens to a conformationally closed pre-fusion state [9]. Interestingly, acidic niches have been reported in T cell-dominated paracortical lymph node zones [84], suggesting that secondary lymphoid microenvironments may be more chemically complex than is widely appreciated. As both LNPs themselves and mRNA-LNP- induced expression are present in lymph nodes [72,85], it is therefore possible that HA conformation could be less uniform in vivo due to heterogeneities in pH within vaccinated tissues. However, such claims remain purely speculative in the absence of evidence showing the presence of these proposed microenvironments and evidence linking these regions with mRNA-LNP vaccinated cells, expressed HA antigens, and HA conformational states.

Screening pCAGGS plasmid-expressed HA antigens in vitro was effective at characterizing mutant HA expression and stability phenotypes. Indeed, nine HA substitutions were identified as meeting expression and stability-based exclusion criteria without requiring the resource- and time-intensive in vitro transcription steps needed to produce functional mRNA (Figure 2 and Figure 3). However, complete recapitulation of mRNA-vaccine transcript phenotypes may be limited by the longer persistence of pCAGGS-expressed proteins than by the persistence of mRNA, potentially skewing expression phenotypes at extended timepoints [59,86]. Conversely, this persistence would enable reliable detection of phenotypes in future sensitive, library-based, high-throughput HA antigen screens. Related deep mutational scanning work has examined HA stability in the context of virus replication [87], although developing clear readouts for this platform at scale would be challenging. However, transduction of “landing pad” sequences into target cells enabled large-scale screening of individual protein phenotypes [88] and could potentially be adapted to screen HA stability in vitro. Additional work is needed to adequately explore these possibilities.

Stalk-mutant mRNA-LNP serology, as well as weight loss and survival when challenged with a drifted Vic/22, had noted similarities to the outcomes observed with WT HA vaccination (Figure 4, Figure 5, Figure 6 and Figure 7). These data are not consistent with the benefits seen by stabilizing the fusion machineries of other structural class 1 fusion proteins [25,26]. Interestingly, multi-location stabilization of the H5 stalk resulted in clear improvements in mRNA-LNP immunogenicity [33]. Indeed, we have also observed measurable, albeit modest, benefits in single-substitution-stabilized HAs [34]. The benefits of HA-stabilizing substitutions might be additive, as well as substitution-specific, and/or subtype-specific. K212C-E216C and E216K mutant vaccines both elicited serological responses similar to those with the WT vaccine, in addition to comparable weight loss and survival after Vic/22 challenge. However, R109E-vaccinated mice, unlike R109C-E69C-vaccinated mice, experienced delayed recovery after Vic/22 challenge that was coincident with undetectable MN titers and diminished total IgG responses (Figure 5J, Figure 6J and Figure 7D). Furthermore, survival of G72K-vaccinated mice challenged with Vic/22 was significantly diminished despite the serology being similar to that of G72D-vaccinated mice. Such substitution-dependent changes in immunogenicity suggest that specific effects at epitopes alter the magnitude of the humoral response. Neither G72D nor G72K overlap with classical epitopes [61], although a direct impact of the G72 amino acid sequence on surface epitopes cannot be excluded. It is possible that G72D and G72K exert indirect structural effects on epitope conformations in a manner similar to those that reduce broadly neutralizing antibody reactivity against central stalk epitopes [89].

Strikingly, E107C-R76C, E107A, and E107R mutant vaccines elicited more HA stalk antibodies than the WT vaccine (Figure 8A,B). After vaccination with E107R and, to a lesser degree, with E107C-R76C and E107A, there were measurable increases in ADCC activity against PR8, Cal/09, HI/19, and, to a lesser extent, Vic/22 (Figure 8C–G). These increases were especially clear in E107R-elicited responses against PR8. However, reductions in E107C-R76C and E107A vaccinated sera ADCC activity against Mich/15 despite WT-like ADCC activity in E107R are suggestive of substitution-dependent differences in Fc-mediated effector function. The reasons explaining these differences are unclear. One possible explanation may be that specific amino acid side chains alter the conformations of local epitopes that are capable of eliciting protection through ADCC activity, ultimately impacting the reactivity of ADCC against specific antigens. However, structural and epitope mapping studies are needed to understand how and why specific modifications at the same location may have differential impacts on ADCC activity. Nevertheless, ADCC responses were more commonly increased in the sera of E107-vaccinated mice, which is consistent with the Fc-mediated effector functions that typify protection imparted by HA stalk antibodies [70]. All three of these substitutions resulted in RBD–RBD domain instability at high pH values (Figure 3G,H,J), but so did E216K, which elicited WT-like responses (Figure 3F). This suggests the importance of residue location to E107 antigen phenotypes. Furthermore, the fact that the HA mRNA-LNP constructs studied here lack a secretion signal likely indicates that E107-associated modifications elicit enhanced stalk responses against membrane-bound proteins. mRNA-LNP vaccines containing chimeric HA antigens that lack a secretion signal have been found to elicit stalk-directed antibody responses [37], demonstrating that engineered HA proteins lacking secretion signals are known to support stalk-directed responses. However, more work is needed to directly assess the exposure of the stalk domain on the surface of a membrane, and how this may impact the antibody response.

In addition to the differences in ADCC activity, there were other substitution-specific differences in the responses elicited by E107 vaccines, when compared with the WT vaccine. Both E107C-R76C- and E107A-vaccinated mice experienced greater weight loss than WT-vaccinated mice when challenged with MA-Cal/09, despite measurable increases in anti-Cal/09 ADCC activity (Figure 8D and Figure 9A,B). The E107C-R76C responses may be explained by the modest decreases in the HI and MN titers against Cal/09, which are suggestive of disruptions to cross-reactive epitopes (Figure 4G and Figure 5G). However, E107A serology was not significantly affected. Monoclonal antibodies that bind to the base of the H3 head cause antibody-dependent enhancement in mice [90]. Given the location of the E107 residue between the head and stalk domains, specific substitutions here could render both the stalk and the base of the head more available for immune recognition. Even so, the survival and viral burden of mice vaccinated with any E107 substitution vaccine and then challenged with Cal/09 were not significantly different from those of WT-vaccinated mice, nor was protection against Vic/22 and PR8 different. Indeed, E107R vaccination imparted WT-like protection against all challenges, with only a small difference in weight loss when mice were challenged with Cal/09 (Figure 7, Figure 8 and Figure 9). A key limitation of this work is the exclusive use of young female mice, which constrains the findings of this paper. Future studies will be needed to explore the antigenic effects of E107-associated modifications in male individuals as well as at-risk populations such as immunocompromised, obese, and pregnant populations.

Additionally, E107R vaccine protection against PR8 was not consistent with elevated ADCC activity, although modest, non-significant increases in post-challenge survival were seen (Figure 9D). The lack of any clear protective advantage imparted by these substitutions supports, but does not confirm, the adequate immunogenicity of WT H1 HA stability in mice after a single immunization. Ultimately, it is premature to attribute the antigenic properties of the mutant mRNA-LNPs studied here, especially E107 mRNA-LNPs, to HA stability without additional structural and immunological evidence to explain the nature and importance of their phenotypes.

In conclusion, we find that E107 residue mutant mRNA-LNPs enhance antibody responses against the subdominant HA stalk domain. Indeed, the E107R phenotype in particular demonstrates that specific H1 HA substitutions may impart antigenic effects in addition to, and not at the expense of, WT-like vaccine immunogenicity. These findings highlight the potential of mutagenic antigen screens to identify modifications that enhance the anti-influenza humoral response, and this work may prove useful in the development of vaccine candidates that aim to elicit HA stalk responses.

## Figures and Tables

**Table 1:** Selection of HI/19 HA stability substitutions for in vitro screening.

H3 Number Scheme	H1 Number Scheme	HA Subunit ^1^	H3/HA2 Residue Numbers ^2^	Domain Interface ^2^	Local Structural Interface ^3^	WT Residue Characteristics ^4^	Predicted Substitution Effect ^5^	Predicted Stability Effect
G72C, K149C	G63C, K146C	HA1, HA1	G72C, K149C	Head	VED Cys-helix-Cys interface with RBD	Cys-helix-Cys α-helix cap, 130–150 loop turn loop [51]	Disulfide bond formation	Stabilize
G72D	G63D	HA1	G72D	Head	VED Cys-helix-Cys interface with RBD	Cys-helix-Cys α-helix cap [51,60]	Add polar interactions	Stabilize
K212C, E216C	K209C, E213C	HA1, HA1	K212C, E216C	Head	RBD–RBD interface^3^ [44]	K212 H-bond with E216 [44]	Disulfide bond formation [44]	Stabilize
G72I	G63I	HA1	G72I	Head	VED Cys-helix-Cys interface with RBD	Cys-helix-Cys α-helix cap [51,60]	Steric clash: K149	Destabilize
G72K	G63K	HA1	G72K	Head	VED Cys-helix-Cys interface with RBD	Cys-helix-Cys α-helix cap [51,60]	Electrostatic repulsion: K149	Destabilize
L108K	L101K	HA1	L108K	Head	VED 110-helix interface with RBD	110-helix residue [51]	Disrupt VED-RBD packing	Destabilize
E216K	E213K	HA1	E216K	Head	RBD–RBD interface^3^ [42]	K212 H-bond with E216 [42,44]	Electrostatic repulsion [42]	Destabilize
R109C, E398C	R109C, E396C	HA1, HA2	R109C, E69C	Head–Stalk	VED 110-helix interface with B-loop(HA2)	110-helix R109 salt bridge with B-loop E69 [49]	Disulfide bond formation	Stabilize
L51C, S270C	L41C, S268C	HA1, HA1	L51C, S270C	Head–Stalk	FD interface with VED	FD, Adjacent to disulfide C52, C-terminal VED residue [51,61]	Disulfide bond formation	Stabilize
L51K, S270D	L41K, S268D	HA1, HA1	L51K, S270D	Head–Stalk	FD interface with VED	FD, adjacent to disulfide C52, C-terminal VED residue [51,61]	Salt bridge formation	Stabilize
E107C, R405C	E100C, R403C	HA1, HA2	E107C, R76C	Head–Stalk	VED 110-helix interface with C-helix(HA2) ^3^	110-helix E107 H-bond with C-helix R76 [43]	Disulfide bond formation	Stabilize
R109G	R102G	HA1	R109G	Head–Stalk	VED 110-helix interface with B-loop(HA2)	110-helix R109 salt bridge with B-loop E69 [49]	Salt bridge elimination [49]	Destabilize
R109E	R102E	HA1	R109E	Head–Stalk	VED 110-helix interface with B-Loop(HA2)	110-helix R109 salt bridge with B-loop E69 [49]	Electrostatic repulsion [49]	Destabilize
I269G	I267G	HA1	I269G	Head–Stalk	FD interface with C-terminal VED	C-terminal VED residue [49,61]	Destabilize VED packing	Destabilize
E107A	E100A	HA1	E107A	Head–Stalk	VED 110-helix interface with C-helix(HA2) ^3^	110-helix E107 H-bond with C-helix R76 [43]	Salt bridge elimination	Destabilize
E107R	E100R	HA1	E107R	Head–Stalk	VED 110-helix interface with C-helix(HA2) ^3^	110-helix E107 H-bond with C-helix R76 [43]	Electrostatic repulsion	Destabilize
H18Q	H8Q	HA1	H18Q	Stalk	Fusion-peptide pocket [27]	HA1 N-terminal noncovalent interactions with M17 [27]	Adds H-bond [27]	Stabilize
G352C	G350C	HA2	G23C	Stalk	One HA1 β-strand in five-strand sheet [51]	β-Turn-β H-bond with HA1 N-terminal G16 [45]	Aid FP packing [45]	Stabilize
H355W	H353W	HA2	H26W	Stalk	Histidine switch region 1 [48]	β-Turn-β H-bond with G-helix K153 [48]	π-Cation interaction [48]	Stabilize
Y448C, E461C	Y446C, E459C	HA2, HA2	Y119C, E132C	Stalk	D-helix(HA2) interface with β-strand-E(HA2)	Polar contact between D-helix and β-strand-E [51]	Disulfide bond formation	Stabilize
N483S	N481S	HA2	N154S	Stalk	G-helix(HA2) [46]	N-glycosylation site [46]	Glycosylation ablation [46]	

^1^ The HA subunit in which the substitution(s) reside(s). Top row for the first residue, bottom row for the second. ^2^ Regional location of the structural interface studied. “Head–Stalk” refers to the interface between these two domains. ^3^ Denotes structural interfaces that exist between two different protomers within the trimer. ^4^ The top row in a cell describes the top residue; the bottom row describes the bottom residue. ^5^ Predicted effect on local structure. Residues are listed if not previously mentioned.

**Table 2:** In vitro phenotypes of HI/19 HA test-set substitutions.

Residue Location	Regional Structural Interface	Total Cell Expression (% of WT) ^1^		% Trypsin Cleavability ^2^		Cell-Surface Expression, (% of WT) ^1,3^		Activation pH by Syncytia Formation ^4^	pH of Conformation Change; 95% CI ^3,5^		Screening Exclusion Status
WT		100		45	±10	100	±8	5.4	5.59;	5.55–5.64	
G72C-K149C	Head	48	±23	14	±7	66	±1	ND	5.56;	5.42–5.63	Exclude
G72D	Head	56	±20	14	±4	84	±4	5.1	5.62;	5.59–5.66	Advance
K212C-E216C	Head	96	±46	23	±14	96	±1	ND	-		Advance
G72I	Head	53	±19	12	±5	68	±1	ND	-		Exclude
G72K	Head	52	±11	12	±4	86	±5	ND	5.42;	5.38–5.45	Advance
L108K	Head	56	±17	12	±5	NE		ND	NE		Exclude
E216K	Head	122	±60	50	±9	92	±3	5.8	6.00;	5.75–6.11	Advance
R109C-E69C	Head–Stalk	88	±60	33	±14	118	±2	ND	-		Advance
L51C-S270C	Head–Stalk	68	±34	28	±11	59	±2	ND	-		Exclude
L51K-S270D	Head–Stalk	80	±41	28	±7	NE		ND	NE		Exclude
E107C-R76C	Head–Stalk	65	±29	29	±9	99	±5	ND	-		Advance
R109G	Head–Stalk	86	±38	34	±9	106	±5	5.4	5.69;	5.59–5.79	Exclude
R109E	Head–Stalk	101	±44	34	±6	108	±5	5.5	5.69;	5.64–5.74	Advance
I269G	Head–Stalk	112	±46	40	±7	94	±3	5.3	5.69;	5.64–5.75	Exclude
E107A	Head–Stalk	141	±54	51	±4	96	±3	5.8	6.34;	6.26–6.59	Advance
E107R	Head–Stalk	59	±24	43	±7	83	±16	5.7	6.11;	5.96–6.57	Advance
H18Q	Stalk	130	±69	52	±9	96	±1	5.0	5.05;	4.36–5.14	Advance
G23C	Stalk	63	±26	28	±7	NE		ND	NE		Exclude
H26W	Stalk	174	±71	52	±6	98	±3	5.1	-		Advance
Y119C-E132C	Stalk	73	±23	23	±6	68	±1	ND	-		Exclude
N154S	Stalk	83	±36	40	±9	105	±4	5.4	5.56;	5.53–5.59	Advance

^1^ Data normalized as a percentage of WT expression and shown as the mean ± SD. ^2^ The percentage of trypsin-cleaved HA is shown as the mean ± SD. ^3^ NE indicates no detectable cell-surface expression. ^4^ Rounded to 0.1 pH units. ND indicates no syncytia formation within the tested pH range. ^5^ pH of 50% increase from baseline to maximum GMF with 95% CI. “-” indicates ambiguous values.

## Data Availability

Any additional data will be made available upon request.

## Supplementary Materials

The following supporting information can be downloaded at https://www.mdpi.com/article/10.3390/vaccines14080701/s1. Figure S1: Flow cytometric gating strategy of HI/19 HA cellular expression in HEK-293T cells; Figure S2: Representative micrographs of syncytia after transfection with pCAGGS-expressed HI/19 HA proteins; Figure S3: Head stability of pCAGGS-expressed HA mutants measured by flow cytometry; Table S1: Wild-Type mRNA-LNP stability characterization.

## Abbreviations

The following abbreviations are used in this manuscript:

ADCC	Antibody-dependent cell-mediated cytotoxicity
AUC	Area under the curve
BSA	Bovine serum albumin
CA/09	A/California/07/2009 virus
Cal/09	A/California/04/2009 virus
cDNA	Complementary DNA
cH6/1	Chimeric hemagglutinin containing H6 head and H1 stalk domains
CI	Confidence interval
COBRA	Computationally optimized broadly reactive antigen
DAPI	4′,6-diamidino-2-phenylindole
FACS	Fluorescence-activated cell sorting
FBS	Fetal bovine serum
FcγRIV	Fc-gamma receptor four
FD	Fusion stalk subdomain of HA1
FP	Fusion peptide
GMF	Geometric mean fluorescence
HA	Hemagglutinin
HAU	Hemagglutination units
Hawaii/19	WT A/Hawaii/70/2019 virus
H-bond	Hydrogen bond
HEK-293T	Human embryonic kidney 293-transformed cells
HI	Hemagglutination-Inhibition
HI/19	A/Hawaii/70/2019 virus
HRP	Horseradish peroxidase
L.O.D	Limit of detection
mAb	Monoclonal antibody
MA-Cal/09	Mouse-Adapted A/California/04/2009 virus
MDCK	Madin–Darby canine kidney cells
Mich/15	A/Michigan/45/2015 virus
MLD_50_	Median lethal dose 50%
MN	Microneutralization titer
mRNA-LNP	Nucleoside-modified messenger RNA lipid nanoparticle vaccine
MWCO	Molecular weight cutoff
NA	Neuraminidase
ND	Not detected
NE	Not expressed
NMWCO	Nominal molecular weight cutoff
PBS^−/−^	Plain phosphate-buffered saline
PBS^+/+^	Phosphate-buffered saline with calcium and magnesium ions
PBST	Plain phosphate-buffered saline with 0.2% Tween 20 detergent
PFU	Plaque-forming unit
pH1N1	2009 pandemic-lineage H1N1 virus
PR8	A/Puerto Rico/8/1934 virus
PVDF	Polyvinylidene difluoride
QIV	Quadrivalent inactivated seasonal influenza vaccine
RBD	Receptor-binding head subdomain of HA1
RDE	Receptor-destroying enzyme
RLU	Relative light unit
RT	Room temperature
SD	Standard deviation
TCID_50_	Tissue culture infectious dose 50%
TMB	3,3′,5,5′-tetramethylbenzidine
TPCK	Tosyl phenylalanyl chloromethyl ketone
VED	Vestigial-esterase head subdomain of HA1
Vic/22	A/Victoria/4897/2022 virus
WT	Wild-type
