# Supplementary material for: An In Vitro Screen Identifies H1 Influenza Hemagglutinin Substitutions That Alter mRNA-LNP Vaccine Responses Against the Stalk Domain
Source: Vaccines (Basel). 2026 Aug 13;14(8):701. doi: 10.3390/vaccines14080701 (PMC13517961; doi:10.3390/vaccines14080701)
Supplement: Supplementary file 1 [file vaccines-14-00701-s001.zip › vaccines-4454529-supplementary.pdf]

Article

An In Vitro Screen Identifies H1 Influenza Hemagglutinin Substitutions that Alter mRNA-LNP Vaccine Responses Against the Stalk Domain

Supplementary data

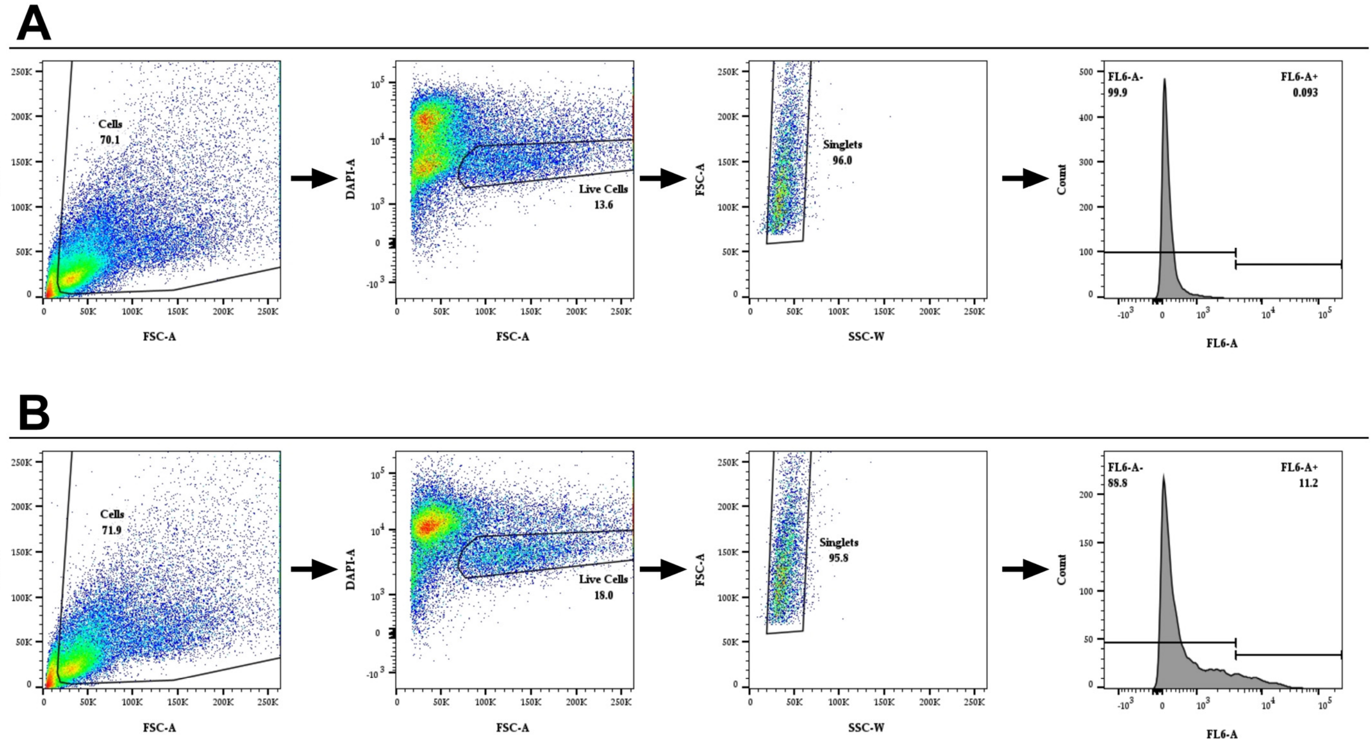

Figure S1. Flow cytometric gating strategy for HI/19 HA cellular expression in HEK-293T cells. Transfected cells were stained as described in the Materials and Methods (subsection 2.6). Cells were first gated into cell-sized subpopulations. The live-cell subpopulation was identified by DAPI staining. From this population, cell singlets were gated into subpopulations for HA expression analysis based on the level of fluorescence. All samples were stained with goat anti-human IgG Alexa Fluor 594 secondary antibody. (**A**) Singlets stained with an isotype control were gated to be negative for fluorescence. (**B**) For each sample, 5000 live single cells were treated with mAb CR6261, using the same strategy as for the isotype control, to identify the HA-expressing subpopulation for the geometric mean fluorescence (GMF) calculation.

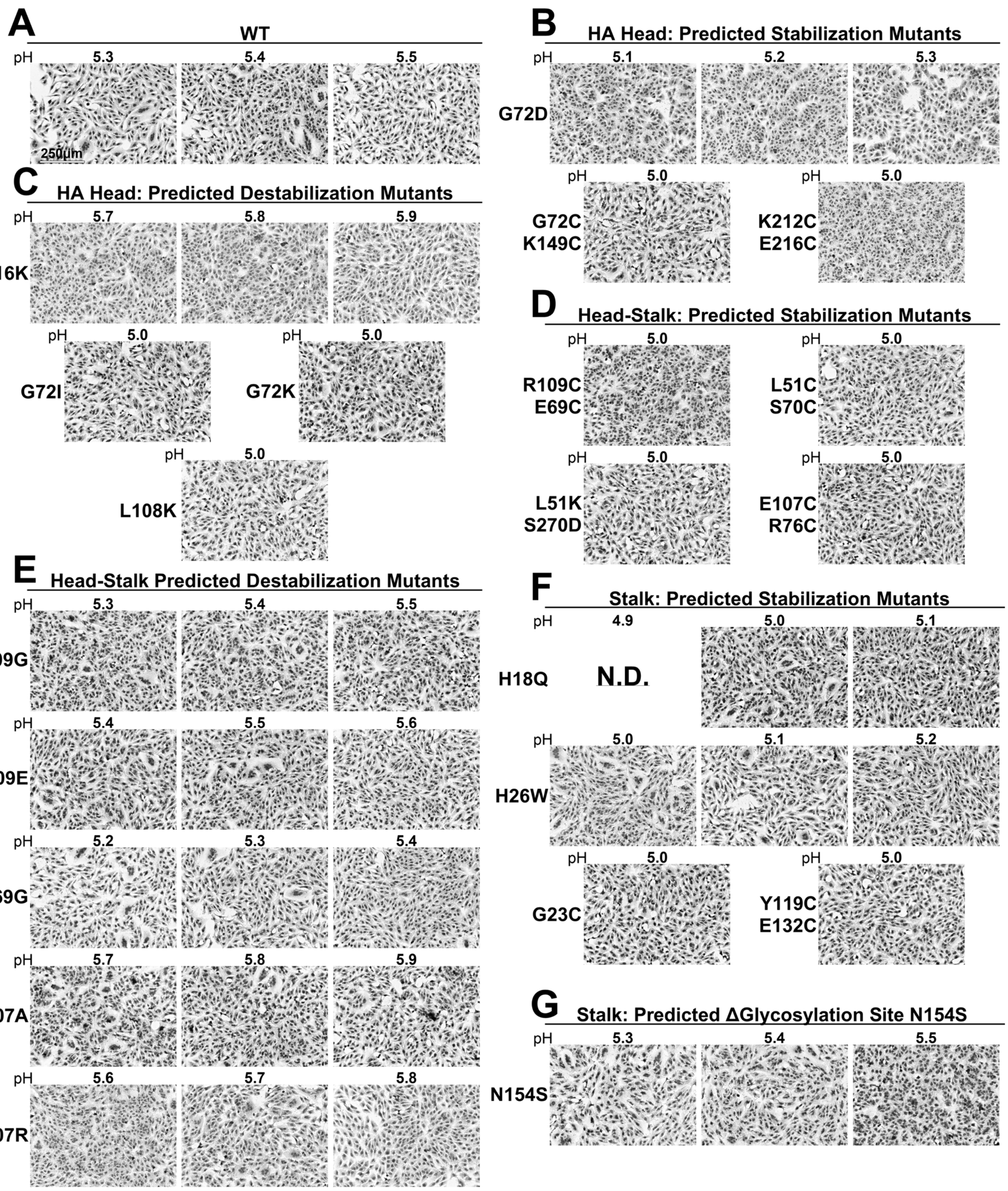

Figure S2. Representative micrographs of syncytia after transfection with pCAGGS-expressed HI/19 HA proteins. Transfected Vero cells were incubated at pHs ranging from 5.0 to 6.0 in intervals of 0.1 pH unit. N.D. (not determined) indicates pH values below the pH 5.0 cutoff. The activation pH was defined as the highest pH value at which syncytia formation was observed. There are three panels for mutants that induced syncytia formation between pH 5.0 and pH 6.0; the middle panel shows the activation pH (the highest pH for syncytia formation). There is one panel for those mutants that did not induce syncytia within this pH range, showing the transfected cells at pH 5.0. (**A**) Syncytia formation with WT HA. (**B**) Syncytia formation by HA mutants predicted to stabilize the head region. (**C**) Syncytia formation by HA mutants predicted to destabilize the head region. (**D**) Syncytia formation by HA mutants predicted to stabilize the head–stalk interface. (**E**) Syncytia formation by HA mutants predicted to destabilize the head–stalk interface. (**F**) Syncytia formation by HA proteins containing HA2 stalk mutations. (**G**) Syncytia formation by the HA-N154S glycosylation site ablation mutant

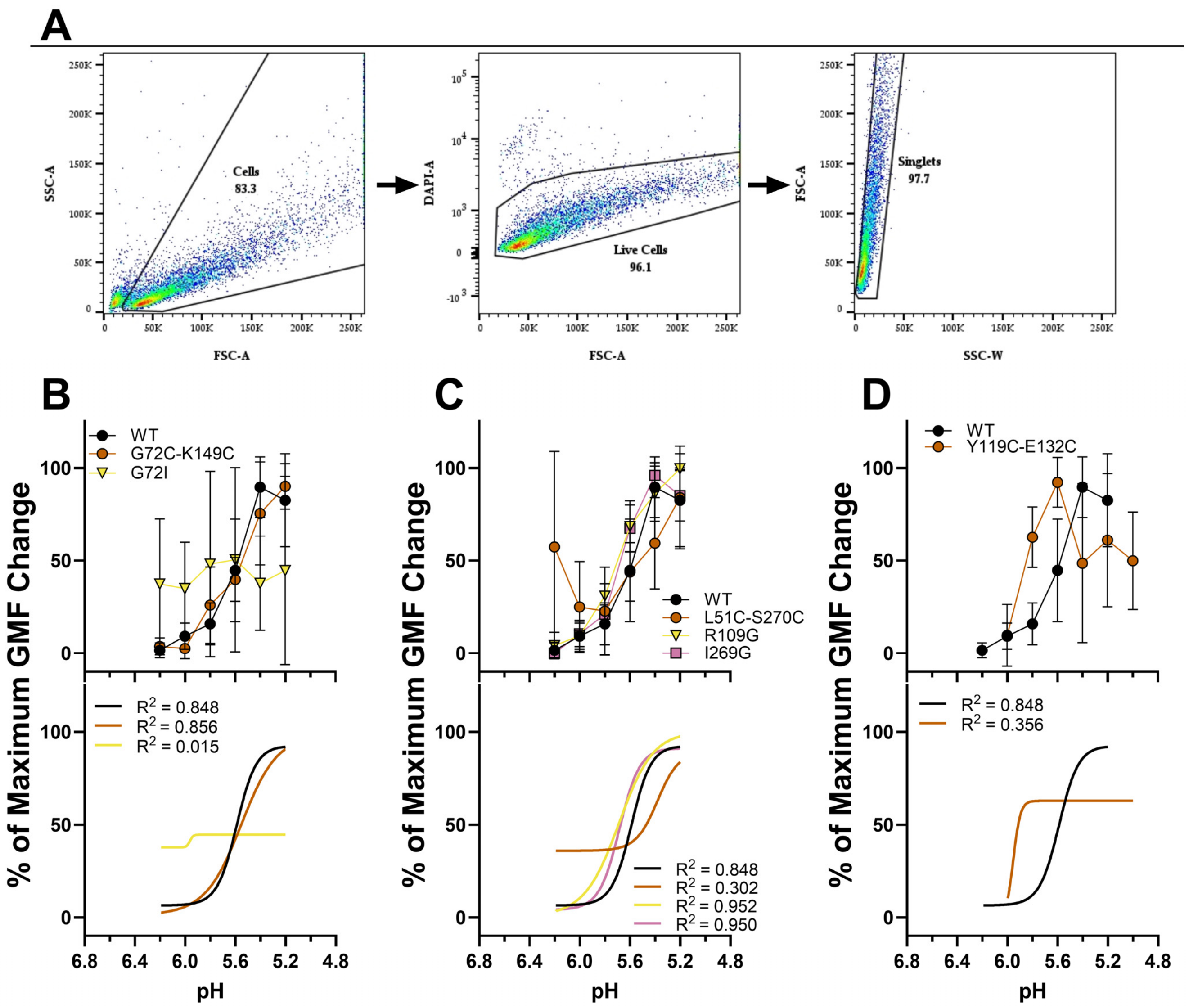

Figure S3. Head stability of pCAGGS-expressed HA mutants measured by flow cytometry. For each HA protein, six transfected cell samples were treated with TPCK trypsin then exposed to decreasing pH from pH 6.6 to pH 5.0. Samples were treated with mAb FluA-20 and stained with Alexa Fluor 594 goat anti-human IgG polyclonal secondary antibody. (**A**) Cells were gated as live single cells with n = 2000 gated cells per sample. Net GMF values were found by subtracting the GMFs of isotype controls from the GMFs of samples. (**B**–**D**) FluA-20 binding of plasmid HA mutants that were subsequently excluded from further examination. The percentage total fluorescence change (maximum GMF − minimum GMF) is displayed as a function of pH. (**B**) Results for head substitutions. (**C**) Results for head–stalk substitutions. (**D**) Results for stalk substitutions.

Table S1. Wild-Type mRNA-LNP Stability Characterization

Vaccine	Temp (°C)	Time Point	Mean Size (nm)	Size SD	n [1]	Mean PDI [2]	PDI SD	n	Mean ZP (mV) [3]	ZP SD	n
WT	4	Baseline	88.13	1.47	3	0.214	0.011	3	1.38	0.34	3
WT	4	2 Weeks	90.11	3.41	3	0.110	0.011	3	1.23	0.28	3
WT	4	1 Month	93.48	2.13	3	0.097	0.004	3	1.44	0.35	3
WT	25	Baseline	90.69	3.48	3	0.219	0.012	3	1.78	0.32	3
WT	25	2 Weeks	91.26	0.90	3	0.111	0.018	3	1.85	0.83	3
WT	25	1 Month	93.37	1.19	3	0.141	0.042	3	2.40	0.39	3

[1] The number of technical replicates

[2] Polydispersity Index (PDI)

[3] Zeta Potential (ZP)
